# A new species of Amazonian snouted treefrog (Hylidae: *Scinax*) with description of a novel species-habitat association for an aquatic breeding frog

**DOI:** 10.7717/peerj.4321

**Published:** 2018-02-09

**Authors:** Miquéias Ferrão, Rafael de Fraga, Jiří Moravec, Igor L. Kaefer, Albertina P. Lima

**Affiliations:** 1 Programa de Pós-Graduação em Ecologia, Instituto Nacional de Pesquisas da Amazônia, Manaus, Amazonas, Brazil; 2 Department of Biological Sciences, Macquarie University, Sydney, Australia; 3 Department of Zoology, National Museum, Prague, Czech Republic; 4 Instituto de Ciências Biológicas, Universidade Federal do Amazonas, Manaus, Amazonas, Brazil; 5 Coordenação de Biodiversidade, Instituto Nacional de Pesquisas da Amazônia, Manaus, Amazonas, Brazil

**Keywords:** Amazonia, Anura, Taxonomy, Ecology, Environmental heterogeneity, Edaphic factors

## Abstract

The genus *Scinax* is one of the most specious genera of treefrogs of the family Hylidae. Despite the high number of potential new species of *Scinax* revealed in recent studies, the rate of species descriptions for Amazonia has been low in the last decade. A potential cause of this low rate may be the existence of morphologically cryptic species. Describing new species may not only impact the taxonomy and systematics of a group of organisms but also benefit other fields of biology. Ecological studies conducted in megadiverse regions, such as Amazonia, often meet challenging questions concerning insufficient knowledge of organismal alpha taxonomy. Due to that, detecting species-habitat associations is dependent on our ability to properly identify species. In this study, we first provide a description of a new species (including its tadpoles) of the genus *Scinax* distributed along heterogeneous landscapes in southern Amazonia; and secondly assess the influence of environmental heterogeneity on the new species’ abundance and distribution. *Scinax ruberoculatus* sp. nov. differs from all nominal congeners by its small size (SVL 22.6–25.9 mm in males and 25.4–27.5 mm in females), by having a dark brown spot on the head and scapular region shaped mainly like the moth *Copiopteryx semiramis* (or a human molar in lateral view, or a triangle), bicolored reddish and grey iris, snout truncate in dorsal view, bilobate vocal sac in males, by its advertisement call consisting of a single pulsed note with duration of 0.134–0.331 s, 10–23 pulses per note, and dominant frequency 1,809–1,895 Hz. Both occurrence and abundance of the new species are significantly influenced by silt content in the soil. This finding brings the first evidence that edaphic factors influence species-habitat association in Amazonian aquatic breeding frogs.

## Introduction

*Scinax* Wagler, 1830 is the second most speciose genus of the family Hylidae Rafinesque, 1815 composed of small to medium sized treefrogs, distributed from Mexico to southern Argentina and Uruguay, including the Caribbean islands Trinidad and Tobago, and Santa Lucia (Frost, 2017). Despite the problematic taxonomic history (see Faivovich, 2002), the genus *Scinax* experienced a nomenclatural stability for almost ten years since Faivovich et al. (2005). Recently, Duellman, Marion & Hedges (2016) revised Hylidae, making considerable changes in *Scinax* (*sensu* Faivovich et al., 2005): the genus *Ololygon* Fitzinger, 1843 was resurrected to harbour the former *Scinax catherinae* Clade (*sensu* Faivovich, 2002), the genus *Julianus* Duellman, Marion & Hedges, 2016 was described to house the former *Scinax uruguayus* species group (*sensu* Faivovich et al., 2005), and the genus *Scinax* is now composed of species of the *Scinax ruber* Clade (*sensu* Faivovich, 2002). Currently, *Scinax* is composed of approximately 71 valid species, while *Ololygon* and *Julianus* are composed by 47 and two species, respectively (Frost, 2017). Furthermore, a new subfamily, Scinaxinae, was described by Duellman, Marion & Hedges (2016) to accommodate the high-supported clade *Scinax* + *Ololygon* + *Julianus* and its sister genus *Sphaenorhynchus* Tschudi, 1838.

Presently, twenty-nine valid species of *Scinax* occur in Amazonia *sensu lato* (Eva & Huber, 2005). However, the number of species occurring in this region (see Table S1) is currently underestimated (Fouquet et al., 2007; Ferrão et al., 2016). An integrative approach combining morphological, bioacoustics and DNA barcoding data revealed existence of seven confirmed candidate *Scinax* species distributed in different parts of the Purus-Madeira Rivers Interfluve (hereafter PMRI) (see Ferrão et al., 2016). Here we describe the confirmed candidate species *Scinax* sp. 7 from Ferrão et al. (2016).

Species are widely recognized as fundamental units in ecology (Gotelli, 2004). Therefore, quantifying species-habitat associations, both at population and assemblage level, is dependent on accurate taxonomic classifications (Isaac, Mallet & Mace, 2004; Bortolus, 2008). Taxonomic inaccuracy may not affect the conclusions of ecological communities studies when a single target taxon is misidentified (e.g., Bortolus, Schwindt & Iribarne, 2002; Bortolus, 2006). However, misidentifying two or more morphologically similar species as a single species may considerably affect the degree of reliance indicated by patterns of species-habitat association (Dexter, Pennington & Cunningham, 2010) because frogs are globally threatened by habitat loss, habitat disconnection, disease and alien species (Kats & Ferrer, 2003; Stuart et al., 2004; Lips et al., 2006; Becker et al., 2007), and some attention have been focused on generating scientific knowledge to support its conservation and management. Species misidentifications should be more common in ecological studies from tropical regions because of the high levels of morphologically cryptic species that co-occur (Kreft & Jetz, 2007; Jenkins, Pimm & Joppa, 2013; Pimm et al., 2014). Therefore, only limited taxonomic data are available for many groups of organisms from tropical rainforests (Giam et al., 2012).

The Amazonia is the largest and most species-diverse tropical rainforest worldwide (Corlett & Primack, 2011). Nonetheless, molecular approaches applied in systematics have revealed that biodiversity in the Amazonia is greatly underestimated by cryptic biodiversity in many groups of organisms, such as frogs (Ron et al., 2012; Jungfer et al., 2013; Caminer et al., 2017). Different species of frogs react differently to the same anthropogenic effect, therefore the taxonomic knowledge is an important key to the conservation of frogs, especially in the Neotropics where the biodiversity is megadiverse. However, the taxonomy and systematics of many frog groups from the Amazonia and the role of environmental variables in shaping the distribution of species remain poorly understood. Furthermore, most taxonomic and ecological studies have been conducted on a local scale; thus, regional approaches are lacking (Allmon, 1991; Tsuji-Nishikido & Menin, 2011; Rojas-Ahumada, Landeiro & Menin, 2012; Dias-Terceiro et al., 2015; Jorge et al., 2016).

Studies that have used frogs to demonstrate patterns of species-habitat association in Amazonia were conducted mostly in the Manaus region (Brazil), on the north bank of the Amazon River (Allmon, 1991; Menin et al., 2007; Menin, Waldez & Lima, 2011; Tsuji-Nishikido & Menin, 2011; Rojas-Ahumada, Landeiro & Menin, 2012; Jorge et al., 2016). In this region, many terrestrial-breeding anurans are influenced by edaphic variables like slope, clay content and pH, while aquatic-breeding anurans are influenced by distance of streams and number of trees (Menin et al., 2007; Menin, Waldez & Lima, 2011; Landeiro, Waldez & Menin, 2014). However, anurans from other parts of Amazonia may present distinct patterns of species-habitat association, once the environmental factors that filter species occurrence and abundance may be expected as a response to regional sets of environmental conditions. As example, the Manaus region exhibits environmental elements that are not observed in the central and northern PMRI, which is the area sampled for this study. This portion of PMRI exhibits forests 17–27 m high, topography relatively flat with local variation from 1 to 3 m, silty soil and shallow water-table (Cintra et al., 2013; Martins et al., 2014; Schietti et al., 2016), whereas the Manaus region is characterized by forests with 30–37 m high closed canopy, undulating topography formed by valleys and plateaux ranging from 40 to 100 m a.s.l., low proportion of silt in the soil, and deep water-table (Ribeiro et al., 1999; Castilho et al., 2006; Schietti et al., 2014).

Reports have indicated that the treefrog genus *Scinax* is adequate for quantifying species-habitat associations because it is an extraordinarily species-rich genus that is widely distributed across different habitats in the Amazonia (Duellman & Wiens, 1993; Fouquet et al., 2007; Ferrão et al., 2016). However, the influence of environmental factors on the species distribution and abundance of *Scinax* is poorly understood, which is likely because of the lack of standardized sampling and the difficulty in identifying the species. Here, we also investigate the influence of environmental variables on the new species’ spatial distribution based on sampling from standardized plots distributed along an approximately 600 km long transect in the PMRI.

## Material and Methods

### Study area

**Figure 1:**
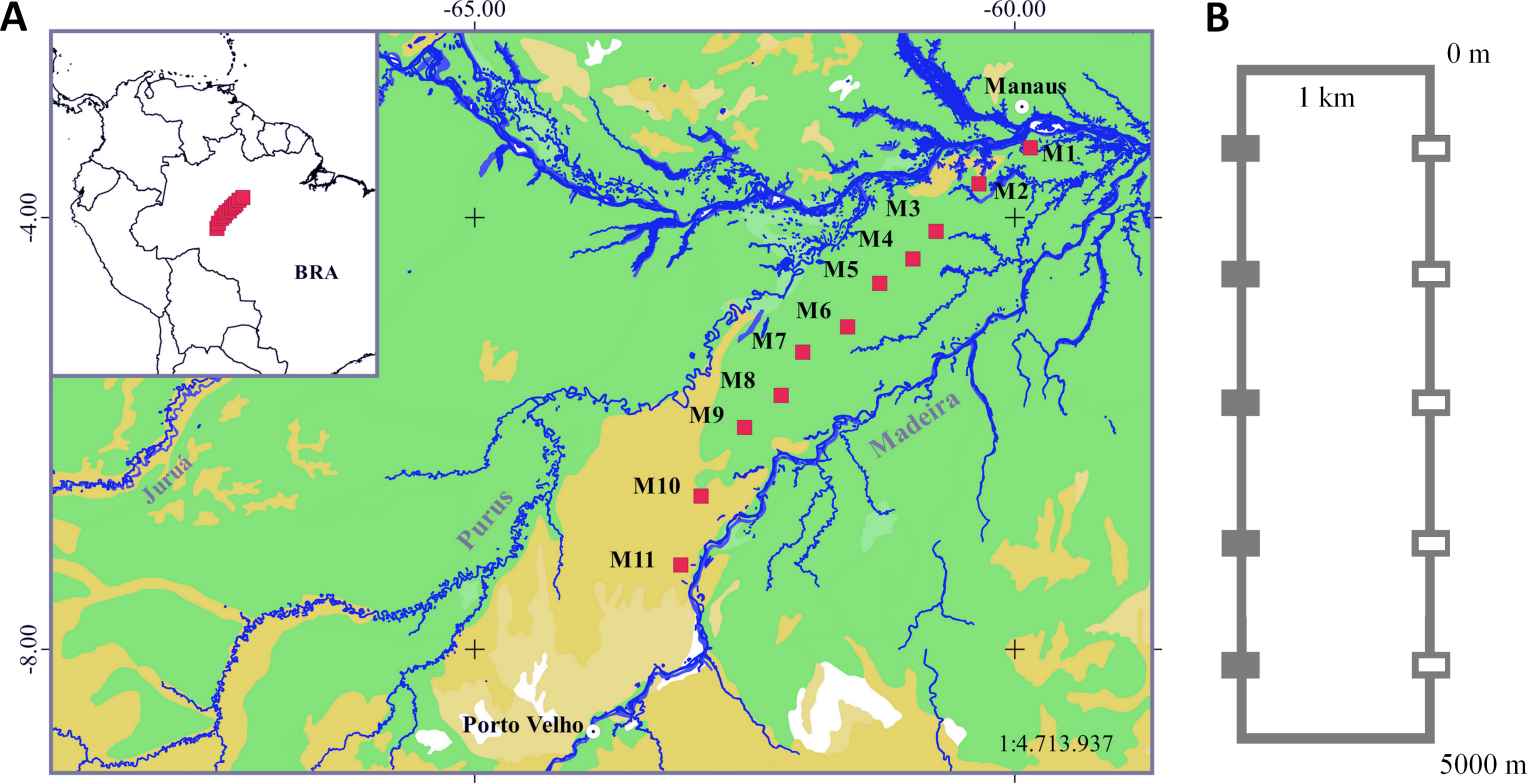
Sampling area in the Purus-Madeira Rivers Interfluve and schematic representation of RAPELD sampling modules and plots. (A) Distribution of RAPELD sampling modules along a 600 km transect. Legend: green colour (M1–M9) = tropical lowland rainforest with emergent canopy; gold colour (M10–M11) = open rainforest lowlands with palm trees. (B) General configuration of each module with ten sampling plots. Open squares represent plots where environmental variables used in this study were measured. BRA, Brazil.

The interfluve between the Purus and Madeira rivers covers approximately 15.4 million hectares of the southern Brazilian Amazonia in an area that is drained by a large and complex stream network (Maldonado et al., 2012). At a broad scale, the northern portion of the PMRI is covered by a tropical lowland rainforest with emergent canopy and the south is covered by open rainforest lowlands with palm trees (IBGE, 1997) (Fig. 1A). At a finer scale, the number of trees and palms in the plots range from approximately 2,000 to 11,500 individuals per hectare (diameter at breast height > 1 cm) and the biomass of plants is lower in plots located in the northern and southern extremes (Schietti et al., 2016). The soil is generally shallow (Martins et al., 2014), with a predominance of silt, followed by sand and clay (Cintra et al., 2013). At a regional scale, the topography of PMRI is relatively flat and the altitude is between 27 and 80 m. Temporary ponds occur in lower areas during the rainy season (Rossetti, Toledo & Góes, 2005) and are formed by local ranges in elevation from 1–3 m.

### Sampling design and collection effort

We collected data from 110 sampling plots (size 250 × 10 m) distributed along a 600 km transect in the PMRI, between the municipalities of Careiro da Várzea (03°11′32″S, 59°52′09″W) and Humaitá (07°13′06″S, 63°05′31″W) (Fig. 1B). The plots were distributed in 11 long-term ecological research sites (hereafter RAPELD) (Magnusson et al., 2013), with approximately 50 km of space between neighbouring modules. Each RAPELD module consists of two parallel 5 km trails and 10 sampling plots, with five plots per trail. The plots were spaced 1 km apart (Fig. 1). The sampling plots followed altitudinal contours to reduce the environmental heterogeneity within each sampling unit (Magnusson et al., 2013).

We sampled *Scinax* specimens using time- and space-constrained visual searches (modified from Campbell & Christman, 1982) and acoustic searches for males in breeding activity. All sampling plots were surveyed by two observers over 90 min. The plots were surveyed three times during the rainy season: in January/February 2013, November 2013, and January/February 2014 (990 sampling hours in total). Although the breeding seasons of most species of *Scinax* are seasonal, and some of them are explosive breeders, the new species was observed breeding along the entire rainy season. Due to that, we do not expect changes in abundance along RAPELD sampling modules caused by our sampling protocol or by sampling sessions. Adults were collected via occasional encounters on access trails and in areas surrounding sampling sites. Tadpoles were encountered in ponds unconnected to streams in the RAPELD module 9, where active breeding adults were encountered.

Adult specimens were killed with a 2% benzocaine solution, fixed in 10% formalin and conserved with 70% ethanol. Tadpoles were killed with a 5% lidocaine solution and conserved in 5% formalin. We used 5% formalin in tadpoles because the labial papillae and jaws are malleable after fixation at that concentration and it facilitates posterior description. Muscular tissue samples of all adults and one tadpole were collected before fixation in formalin, and preserved in absolute ethanol. Adults and tadpoles were deposited in the herpetological section of the Zoological Collections of the Instituto Nacional de Pesquisas da Amazônia (INPA-H), Manaus, Brazil. Tissue samples were deposited in the Albertina Pimentel Lima’s Laboratory at Instituto Nacional de Pesquisas da Amazônia (INPA). See Appendix S1 for the specimens used in the comparisons.

Specimens were collected from RAPELD sampling modules under permit of Instituto Chico Mendes de Conservação da Biodiversidade (ICMBio) and Centro Nacional de Pesquisa e Conservação de Répteis e Anfíbios (RAN) Number 13777. ICMBio and RAN are institutes of Ministry of Environment, Government of Brazil. These permits were subject to approval of all procedures for collecting and euthanizing frogs.

### Morphometric and acoustic data

Morphometric data were collected for adults using a digital calliper, and measurements were performed to the nearest 0.1 mm. Small measurements were taken with a digital calliper under a stereomicroscope. Specimens were sexed through the presence or absence of vocal sac, nuptial pads, vocal slits, and/or eggs. Males were defined as sexually active adults by the observation of expanded vocal sacs. Females were classified as adults when eggs were observed inside the body cavity, or when they were collected in amplexus with or near active males in temporary ponds. All males and females analysed in this study were classified as adults. Sixteen taxonomically important traits for identifying *Scinax* were measured as explained bellow (see Appendix S3 for measurement data). Ten morphometric characteristics were measured according to Duellman (1970): snout-vent length (SVL), head length (HL), head width (HW), horizontal eye diameter (ED), upper eyelid width (UEW), internarial distance (IND), interorbital distance (IOD), horizontal tympanum diameter (TD), tibia length (TL) and foot length (FL). Three characteristics were measured according to Napoli (2005): eye-nostril distance (END), fourth finger disk diameter (4FD), and fourth toe disk diameter (4TD). The length of the tarsus (TAL), hand (HAL) and thigh (THL) followed Heyer et al. (1990). The webbing formulae follow Savage & Heyer (1967) as modified by Myers & Duellman (1982). The enumeration of fingers followed Fabrezi & Alberch (1996) that demonstrated the loss of finger I in the anuran evolution. Description of the colour in preservative is based on all paratypes and given in percentage (between parentheses). Colour in life is described based on photographs of five specimens (except the colour of iris, which was denoted in all paratypes).

The identity of the tadpoles was confirmed by Ferrão et al. (2016) through two distinct molecular barcoding approaches: Automatic Barcoding Gap Discovery—ABGD (Puillandre et al., 2012) and Generalized Mixed Yule Coalescent—GMYC (Pons, Barraclough & Gomez-Zurita, 2006). The 16S rRNA sequence under GenBank accession number KU317405 included in the analyses of Ferrão et al. (2016) was obtained from tissue sample of one tadpole of the lot INPA-H 35410. The developmental stages of the tadpoles were classified according Limbaugh & Volpe (1957), as modified by Gosner (1960). Fourteen morphometric characteristics of 13 tadpoles (stages 34–39) were measured under a stereo-microscope. The terminology and methods of seven measurements followed Altig & McDiarmid (1999): total length (TL), body length (BL), interorbital distance (IOD), tail length (TAL), maximum width of the tail muscle (TMW), maximum height of the tail (MTH), and maximal tail muscle height (TMH). Seven measurements followed Randrianiaina et al. (2011): body width (BW), body height (BH), eye diameter in dorsal view (ED), length of the spiracle (SL), vent tube length (VL), rostro-narial distance (RN), naris-pupil distance (NP).

We used a Principal Components Analysis (PCA) to detect sexual dimorphism in the morphometric data by visually checking for overlapping individuals in the morphometric multivariate space. The PCA was performed using the SVL and 15 morphometric ratios (measurement/SVL) from 28 males and 6 females. Because the SVL and ratios have different scales, we used the command line “*scale = TRUE*” in the *prcomp* function of the R platform (R Core Team, 2016). We also used a Multivariate Analysis of Variance (MANOVA) to test for significant differences between the PCA scores by sex. The first two Principal Components (PCs) of the PCA were used as dependent variables, and sex was used as a factor in the MANOVA model implemented in the R platform (R Core Team, 2016).

Because of the wide distribution of the new species along the PMRI, we verified whether morphological variation occurred in males and females across the RAPELD modules. Given that no significant morphological dimorphism was found between males and females (see results), we performed a MANOVA where the first two components of the PCA quoted above were used as dependent variables and RAPELD sampling modules were used as a factor.

The advertisement call of one male was recorded using a PMD 660 digital recorder (Marantz, Kanagawa, Japan) and a ME 66 directional microphone (Sennheiser, Wedemark, Germany). The calls were analysed using oscillogram and spectrogram (Blackman window, 80 Hz of frequency resolution and 1,024-point Fast Fourier Transform) generated using Raven 1.5 software (Bioacoustics Research Program, 2014). Terminology of acoustic parameters following Köhler et al. (2017). As the advertisement call of the new species consists of a single note, we considered this unit a call (see Köhler et al., 2017). The following spectral and temporal parameters were obtained from 20 calls of the recorded male: call duration (s), number of pulses per call, pulse duration, inter-pulse interval, pulse repetition rate (pulse/s), and minimum, maximum and dominant frequency (Hz) of the call.

### Species-habitat association

Given that the term “habitat” has different concepts and confusion over its use can be result of ambiguity, we follow the definition by Block & Brennan (1993): “the subset of physical environmental factors that a species requires for its survival and reproduction”.

We selected three predictor variables to investigate the influence of the environment on the distribution and abundance of the new *Scinax* species from the PMRI: (1) Forest structure, which was represented by the number of trees (diameter at breast height > 1 cm). We selected tree density to represent forest structure because this variable has been identified as an important factor affecting the distribution and abundance of frogs with aquatic reproduction in Amazonia (Menin, Waldez & Lima, 2011; Landeiro, Waldez & Menin, 2014). (2) Soil structure, which was represented by the percentage of silt because the soil texture in the study area consists primarily of silt (Cintra et al., 2013). Additionally, silty soils are structurally unstable, which increases water retention (Juo & Franzluebbers, 2003). (3) Depth of underground water, which was selected because shallow underground water can overflow lower areas of the study transect. Both soil structure and depth of underground water are variables directly related to the water availability in the pond, and indirectly related to the breeding preferences of the new species, which deposits its eggs only in ponds not connected to streams. Although the distance of stream is a variable commonly used to explain the distribution of aquatic breeding frogs in Amazonia, we do not evaluate it because the reproduction of the new species occurs only in ponds not connected to lotic water bodies. See Appendix S2 for sampling methods related to predictor variables.

Our standardized sampling procedures registered a higher number of *Scinax* individuals compared with previous studies conducted in the Brazilian Amazonia (e.g., Ribeiro, Lima & Magnusson, 2012). However, the number of recorded specimens was low. Hence, we used the sum of recorded individuals (instead of the mean) for the three sampled periods to represent the abundance of *Scinax* in each plot (Bueno et al., 2012). Occasional encounters were not included in the ecological analyses described below.

The normality of the data was assessed using the Shapiro–Wilk test (*P* < 0.05 in all cases). The soil and forest structure data were normalized using log(*x*), and the underground water and frog abundance data were normalized with log(*x* + 1). We tested for correlations among the environmental variables using Spearman’s coefficient. The environmental variables were not correlated to each other. We investigated the effects of the predictor variables on the distribution (presence/absence) of the new species of *Scinax* using the inflated zero regression model (Zeileis, Kleiber & Jackman, 2008), which was run using the R-package pscl (Jackman, 2015). The effect of each predictor variable on the abundance of the new species was tested using simple linear regression. The significance level in regression models and zero-inflated model was *α* = 0.05

### Taxonomic statement

The electronic version of this article in Portable Document Format (PDF) will represent a published work according to the International Commission on Zoological Nomenclature (ICZN), and hence the new names contained in the electronic version are effectively published under that Code from the electronic edition alone. This published work and the nomenclatural acts it contains have been registered in ZooBank, the online registration system for the ICZN. The ZooBank LSIDs (Life Science Identifiers) can be resolved and the associated information viewed through any standard web browser by appending the LSID to the prefix http://zoobank.org/. The LSID for this publication is: urn:lsid:zoobank.org:pub:D50A0044-0EA6-4619-B0FF-42A7443C4EAB. The online version of this work is archived and available from the following digital repositories: PeerJ, PubMed Central and CLOCKSS.

## Results

### Taxonomic account

**Table:** 

**Figure 2:**
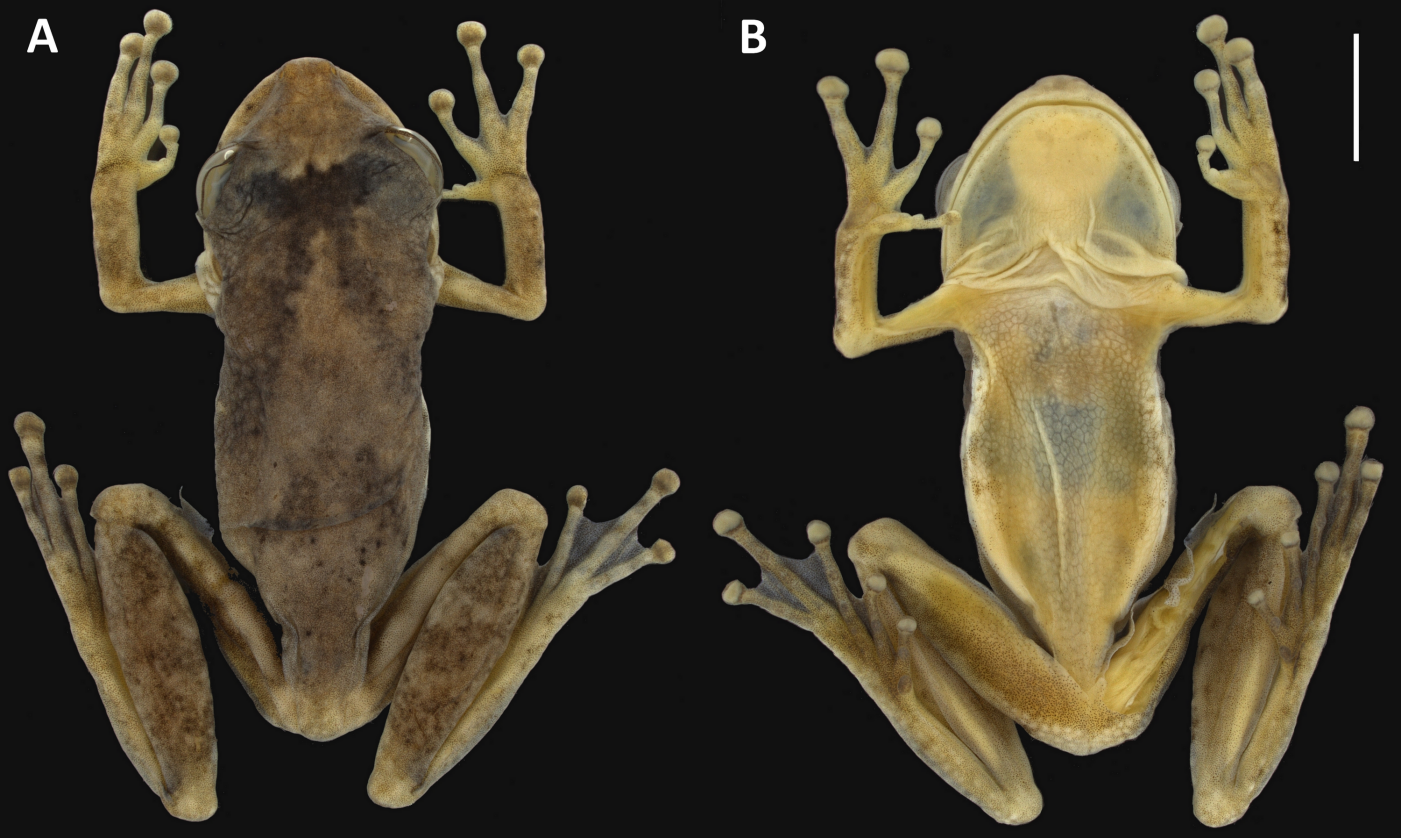
Holotype of *Scinax ruberoculatus.* sp. nov. INPA-H 34665. (A) Dorsal view. (B) Ventral view. Scale = 5 mm. Photos by M Ferrão.

**Figure 3:**
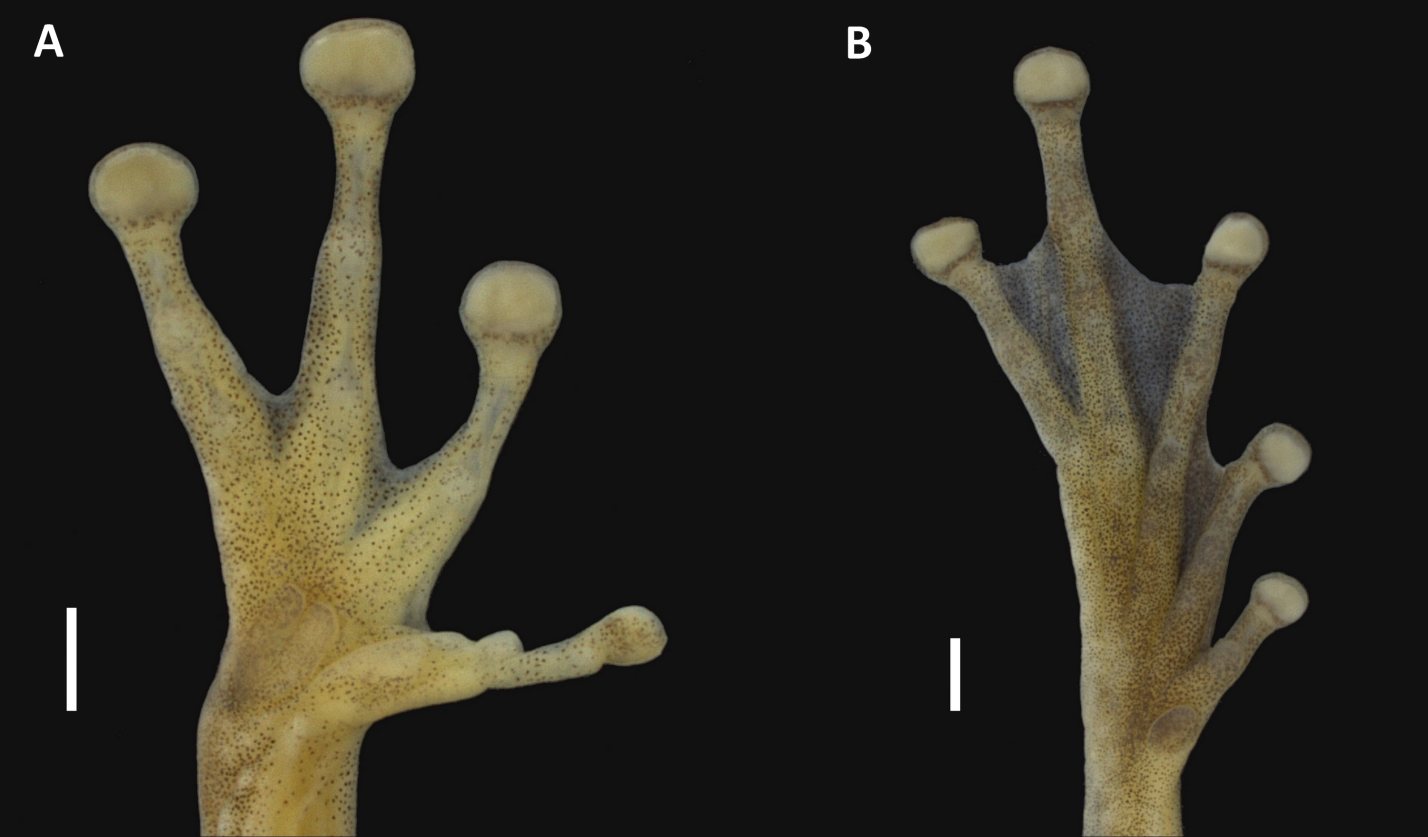
Hand and foot of the holotype of *Scinax ruberoculatus* sp. nov. INPA-H 34665. Scale = 1 mm. Photos by M Ferrão.

*Scinax ruberoculatus* sp. nov.
*Scinax* sp. 7 Ferrão et al. (2016), p. 7, 9, Figs. 2–3.

*Holotype*. INPA-H 34665 (Figs. 2 and 3), adult male collected by Miquéias Ferrão on 18 November 2013, from RAPELD sampling module 9 at kilometre 450 of the BR-319 federal highway (5°56′40″S, 62°30′04″W; 68 m a.s.l.; WGS84), Nascentes do Lago Jari National Park, municipality of Tapauá, Amazonas State, Brazil.

*Paratopotypes*. Three specimens: two adult males (INPA-H 34613, 34619) and one adult female (INPA-H 34605) collected by Miquéias Ferrão and Rafael de Fraga in January and November 2013.

*Paratypes*. Thirty specimens, all collected in Amazonas State, Brazil. Ten: one adult female (INPA-H 34600) and nine adult males (INPA-H 34601, 34604, 34614, 34615, 34622, 34598, 34624, 34627, 34629) collected by Miquéias Ferrão and Rafael de Fraga in February 2011, March 2013, and February 2014 from the RAPELD sampling module 2 at km 100 of the BR-319 federal highway (3°40′40″S, 60°18′56″W; 43 m a.s.l.; WGS84), municipality of Careiro da Várzea. One: an adult male (INPA-H 34602) collected by Pedro Leitão on 18 January 2011 from RAPELD sampling module 3 at km 168 of the BR-319 federal highway (4°8′40″S, 60°43′20″W; 39 m a.s.l.; WGS84), municipality of Careiro da Várzea. Two: one adult male (INPA-H 34610) and one adult female (INPA-H 34620) collected by Miquéias Ferrão in February 2014 from RAPELD sampling module 4 at km 220 of the BR-319 federal highway (4°23′26″S, 60°56′11″W; 47 m a.s.l.; WGS84), municipality of Borba. One: an adult male (INPA-H 34608) collected by Rafael de Fraga on 29 January 2013 from RAPELD sampling module 5 at km 260 of the BR-319 federal highway (4°36′34″S, 61°15′00″W; 50 m a.s.l.; WGS84), municipality of Beruri. Twelve: two adult females (INPA-H 34607, 34630) and ten adult males (INPA-H 34599, 34609, 34611, 34612, 34617, 34618, 34621, 34625, 34626, 34628) collected by Miquéias Ferrão and Rafael de Fraga in January 2013, November 2013, and January 2014 from RAPELD sampling module 7 at km 350 of the BR-319 federal highway (5° 15′57″S, 61°55′58″W, 59 m a.s.l.; WGS84), Igapó-Açú Sustainable Development Reserve, municipality of Beruri. Four: three adult males (INPA-H 34603, 34616, 34623) and one adult female (INPA-H 34606), collected by Miquéias Ferrão and Rafael de Fraga in January 2013, November 2013, and January 2014 from RAPELD sampling module 8 at km 400 of the BR-319 federal highway (5°37′31″S, 62°10′56″W, 67 m a.s.l.; WGS84), Lago do Capanã Grande Extractive Reserve, municipality of Manicoré.

*Etymology.* The specific epithet *ruberoculatus* is composed of two words in Latin, “ruber” (red) and “oculatus” (having eyes). The name is an adjective in concordance with the masculine gender of the genus *Scinax* and refers to the reddish colour of the upper part of the iris. Suggested English common name: ‘Red-eyed snouted treefrog’.

*Generic placement.* We assign the new species to *Scinax* (*sensu* Duellman, Marion & Hedges, 2016) based on general morphological similarity to other members of the genus, cloacal tube of tadpoles positioned above the margin of the lower fin (a putative synapomorphy of the former *S. ruber* Clade according Faivovich et al., 2005), and our previous molecular data (Ferrão et al., 2016).

*Diagnosis*. A small species of the genus *Scinax* characterized by the following combination of characteristics: SVL 22.6–25.9 mm in males and 25.4–27.5 mm in females; snout truncate in dorsal view and rounded in lateral view; tarsal tubercles indistinct; tubercles on the lower jaw, knee, and heel absent; diameter of disc on fourth finger represents 60% of tympanum diameter; skin on dorsum smooth; dentigerous processes of vomers triangular; bilobate vocal sac and nuptial pads in males; Finger III<V; in life, ground colour of dorsum light grey or light brown; a large brown or grey spot on the head and scapular region shaped like the moth of the species *Copiopteryx semiramis* (Cramer, 1775), or a human molar in lateral view, or a triangle; dorsal or dorsolateral stripes absent; whitish cream stripe in the lower portion of the flanks; anterior and posterior surfaces of thighs brown; webbing between toes light to dark grey; belly white to greyish-white with light brown to brown blotches laterally; males with vocal sac semi-translucent white; iris bicolored, upper half reddish, lower half grey; advertisement call consisting of a single pulsed note, with note duration of 0.134–0.331 s, 10–23 pulses/note, dominant frequency 1809–1895 Hz; tadpoles with labial teeth formula 2 (2)/3, absence of labial arm, and presence of dark brown blotch on the distal part of the tail.

*Comparison*. Currently, the genus *Scinax sensu* Duellman, Marion & Hedges (2016) includes 71 species (Frost, 2017). Morphologically, *Scinax ruberoculatus* sp. nov. can be distinguished from all these species and from six confirmed candidate species identified by Ferrão et al. (2016) by following combination of characters (characters of other species in parentheses or brackets unless otherwise stated).

The new species differs from *S*. *boulengeri* (Cope, 1887), *S. constrictus* Lima, Bastos & Giaretta, 2005, *S. garbei* (Miranda-Ribeiro, 1926), *S. jolyi* Lescure & Marty, 2000, *S. kennedyi* (Pyburn, 1973), *S. nebulosus* (Spix, 1824), *S. pedromedinae* (Henle, 1991), *S. proboscideus* (Brongersma, 1933), *S. rostratus* (Peters, 1863), and *S. sugillatus* (Duellman, 1973) by the absence of tubercles on the lower jaw and on knee, and by the absence of an elongated or pointed snout (tubercles on the lower jaw and on knee present, snout elongated or pointed; Duellman, 1972a; Duellman, 1973; Duellman & Wiens, 1992; Lescure & Marty, 2000; Lima, Bastos & Giaretta, 2005). Furthermore, tadpoles of *S. ruberoculatus* sp. nov. differ from tadpoles of *S. boulengeri*, *S. garbei*, *S. nebulosus*, *S. pedromedinae*, *S. rostratus*, *S. sugillatus* by the absence of labial arm and by presence of dark brown blotch on the distal portion of the tail (labial arm present, blotch on the distal portion of the tail absent; Duellman, 1978; McDiarmid & Altig, 1990; Hero & Mijares-Urrutia, 1995; Duellman, 2005; Gomes, Alves & Peixoto, 2014).

The small body size of the males (SVL 22.6–25.9 mm) distinguishes *S. ruberoculatus* sp. nov from the following species: *S. acuminatus* (Cope, 1862) (SVL 39.0–45.0 mm; Lutz, 1973); *S. baumgardneri* (Rivero, 1961) (SVL 29.0–32.0 mm; Rivero, 1961); *S. blairi* (Fouquette & Pyburn, 1972) (SVL 27.8–30.1 mm; Fouquette & Pyburn, 1972); *S. boesemani* (Goin, 1966) (SVL 28.4–31.8 mm; Duellman, 1986); *S. camposseabrai* (Bokermann, 1968) (SVL 28.9–33.5 mm; Caramaschi & Cardoso, 2006); *S. castroviejoi* De la Riva, 1993 (SVL 45.0 mm; De la Riva, 1993); *S. chiquitanus* (De la Riva, 1990) (SVL 27.9–33.3 mm; Duellman & Wiens, 1993); *S. dolloi* (Werner, 1903) (male syntype SVL 34.9 mm; according Araujo-Vieira, Brandão & Faria, 2015); *S. elaeochrous* (Cope, 1875) (SVL 26–32 mm; Savage, 2002); *S. eurydice* (Bokermann, 1968) (SVL 44.0–52.0 mm; Bokermann, 1968); *S. funereus* (Cope, 1874) (SVL 29.8–36.9 mm; Duellman, 1971; Duellman & Wiens, 1993); *S. fuscovarius* (Lutz, 1925) (SVL 41–44 mm; Cei, 1980); *S. granulatus* (Peters, 1871) (SVL 32.0–38.0 mm; Cei, 1980); *S. haddadorum* Araujo-Vieira, Valdujo & Faivovich, 2016 (SVL 29.4–35.4 mm; Araujo-Vieira, Valdujo & Faivovich, 2016); *S. hayii* (Barbour, 1909) (SVL 39.0–42.0 mm; Lutz, 1973); *S. ictericus* Duellman & Wiens, 1993 (SVL 26.3–31.8 mm; Duellman, 2005); *S. iquitorum* (Moravec et al., 2009) (SVL 35.0–38.5 mm; Moravec et al., 2009); *S. manriquei* Barrio-Amorós, Orellana & Chacón-Ortiz, 2004 (SVL 27.7 mm; Barrio-Amorós, Orellana & Chacón-Ortiz, 2004); *S. maracaya* (Cardoso & Sazima, 1980) (SVL 26.7–28.0 mm; Cardoso & Sazima, 1980); *S. nasicus* (Cope, 1862) (SVL 27–35 mm; Cei, 1980); *S. oreites* Duellman & Wiens, 1993 (SVL 28.4–33.5 mm; Duellman & Wiens, 1993); *S. perereca* Pombal Jr, Haddad & Kasahara, 1995 (SVL 34.0–38.5 mm; Pombal Jr, Haddad & Kasahara, 1995); *S. quinquefasciatus* (Fowler, 1913) (SVL 29.6–34.0 m; Duellman, 1971); *S. rossaferesae* Conte et al., 2016 (SVL 27.8–31.6 mm; Conte et al., 2016); *S. ruber* (Laurenti, 1768) (SVL 29.4–41.2 mm; Duellman & Wiens, 1993), *S. sateremawe* Sturaro & Peloso, 2014 (SVL 35.2–38.1 mm; Sturaro & Peloso, 2014); *S. similis* (Cochran 1952) (SVL 30.4–36.8 mm; Juncá et al., 2015); *S. tigrinus* Nunes, Carvalho Jr & Pereira, 2010 (SVL 28.4–30.8 mm; Nunes, Carvalho Jr & Pereira, 2010), and *S. x-signatus* (Spix, 1824) (SVL 32.4–38.7 mm; Juncá et al., 2015). In the PMRI, males of the new species are smaller than males of *Scinax onca* Ferrão et al., 2017 (SVL 31.3–34.3 mm; Ferrão et al., 2017), *Scinax* sp. 5 (SVL 29.6–33.9 mm; Ferrão et al., 2016).

Males of *S. ruberoculatus* sp. nov. are larger than those of *S. exiguus* (Duellman, 1986) (SVL 18.0–20.8 mm; Duellman, 1986).

The body size of females (SVL 25.4–27.5 mm) of *S. ruberoculatus* sp. nov. differs from those of *S. cabralensis* Drummond, Baêta & Pires, 2007 (SVL 24.2–25.1 mm; Drummond, Baêta & Pires, 2007), *S. cretatus* Nunes & Pombal Jr, 2011 (SVL 29.2–32.5 mm; Nunes & Pombal Jr, 2011), *S. curicica* Pugliese, Pombal Jr & Sazima, 2004 (SVL 28.5–31.5 mm; Pugliese, Pombal Jr & Sazima, 2004), *S. danae* Duellman, 1986 (SVL 27.8–29.5 mm; Duellman, 1986), *S. imbegue* Nunes, Kwet & Pombal Jr, 2012 (SVL 28.8–38.0 mm; Nunes, Kwet & Pombal Jr, 2012), *S. montivagus* Juncá et al., 2015 (SVL 28.9–32.2 mm; Juncá et al., 2015), *S. rogerioi* Pugliese, Baêta & Pombal Jr, 2009 (SVL 28.0–34.5 mm; Pugliese, Baêta & Pombal Jr, 2009).

The absence of distinct dorsolateral stripe and the presence of a dark brown spot on the head/scapular region shaped like the moth species *C. semiramis* (or like a human molar in lateral view, or a triangle in living specimens) differ the new species from *S. altae* (Dunn, 1933) (longitudinal dorsolateral stripes present; Savage, 2002), *S. auratus* (Wied-Neuwied, 1821) (light dorsolateral stripes present; Santana et al., 2009), *S. caldarum* (Lutz, 1968) (longitudinal stripes on the back present; Lutz, 1973), *S. cardosoi* (Carvalho-e Silva & Peixoto, 1991) (dorsolateral stripes present; Carvalho-e Silva & Peixoto, 1991), *S. fuscomarginatus* (Lutz, 1925) (divergent or parallel dorsolateral stripes present; Brusquetti et al., 2014), *S. madeirae* (Bokermann, 1968) (convergent dorsolateral stripes present; Brusquetti et al., 2014), *S. pachycrus* (Miranda-Ribeiro, 1937) (dorsolateral stripes present; Lutz, 1973), *S. villasboasi* Brusquetti et al., 2014 (divergent dorsolateral stripes present; Brusquetti et al., 2014), *S. rupestris* (scattered small round and irregular dark blotches on dorsum: Araujo-Vieira, Brandão & Faria, 2015), *S. squalirostris* (Lutz, 1925) (double black lateral bands divided by a white interspace; Lutz, 1973), *S. tymbamirim* Nunes, Kwet & Pombal Jr, 2012 (internal and external dark brown stripes border dorsolateral white stripes; Nunes, Kwet & Pombal Jr, 2012), and *Scinax* sp. 2 *sensu* Ferrão et al. (2016) (dark brown dorsolateral stripes present; Ferrão et al., 2016).

The number of pulses (10–23) in the advertisement call differs *Scinax ruberoculatus* sp. nov. from *S. alter* (Lutz, 1973) (29–152 pulses; Pombal Jr, Bastos & Haddad, 1995; Nunes, Kwet & Pombal Jr, 2012), *S. crospedospilus* (Lutz, 1925) (5–7 pulses; Magrini et al., 2011), *S. cuspidatus* (Lutz, 1925) (5–6 pulses; Pombal Jr, Bastos & Haddad, 1995, *S. duartei* (Lutz, 1951) (4–8 pulses; Pugliese, Pombal Jr & Sazima, 2004), and *S. juncae* Nunes & Pombal Jr, 2010 (4–5 pulses; Nunes & Pombal Jr, 2010).

The smooth dorsal skin differs *Scinax ruberoculatus* sp. nov. differs of from *S. staufferi* (Cope, 1865) (tuberculate: Savage, 2002).

The new species differs from *S. cruentomma* (Duellman 1972) by having 10–23 pulses in the advertisement call (39–54 pulses; Carvalho et al., 2015), upper iris red and lower iris grey (horizontal red bar in the iris; Duellman, 1972b), diameter of disc on fourth finger represents 60% of tympanum diameter (diameter of disc equal to tympanum diameter; Duellman, 1972b), presence of nuptial pad and bilobate vocal sac in males (absence of nuptial pad, and simple vocal sac; Duellman, 1972b), labial teeth formula 2 (2)/3 in tadpoles (2 (2)/3 (1); Duellman, 1972b) and presence of dark brown blotch on the distal part of the tail (absence; Duellman, 1972b).

*Scinax ruberoculatus* sp. nov. can be distinguished from *S. lindsayi* Pyburn, 1992 by the snout truncate in dorsal view (rounded; Pyburn, 1992), presence of nuptial pad in males (absence; Pyburn, 1992), Finger III<V (Finger III = V; Pyburn, 1992), diameter of disc on fourth finger 60% of tympanum diameter (diameter of disc equal to tympanum diameter; Pyburn, 1992), call duration 0.134–0.331 s (0.08–0.10 s; Pyburn, 1992), upper iris reddish and lower iris grey (iris pinkish bronze; Pyburn, 1992).

The new species can be differentiated from *S. wandae* (Pyburn & Fouquette, 1971) by 10–23 pulses in the advertisement call (70–108 pulses; Pombal Jr et al., 2011), absence of dorsolateral dark brown stripes (presence; Pyburn & Fouquette, 1971), diameter of disc on fourth finger represents 60% of tympanum diameter (diameter of disc equal to tympanum diameter; Pyburn & Fouquette, 1971), tongue lanceolate (rounded; Pyburn & Fouquette, 1971), vocal sac smooth (finely granular; Pyburn & Fouquette, 1971).

*Scinax ruberoculatus* sp. nov. can be distinguished from *S. karenanneae* (Pyburn, 1973) by having snout truncate in dorsal view (rounded; Pyburn, 1993), SVL 22.6–25.9 mm in males (SVL 26.6–28.9 mm; Pyburn, 1993); presence of nuptial pad in males (absence; Pyburn, 1993), Finger III<V (Finger III = V; Pyburn, 1993), diameter of disc on fourth finger 60% of tympanum diameter (diameter of disc about equal to tympanum diameter; Pyburn, 1992), males with vocal sac semi-translucent white (yellow; Pyburn, 1993), upper iris red and lower iris grey (bright golden bronze; Pyburn, 1993).

The bicolored iris (reddish upper and grey lower) distinguishes *Scinax ruberoculatus* sp. nov. from *Scinax* sp. 1 (red horizontal band in the central portion of the iris; Ferrão et al., 2016), *Scinax* sp. 4 (red horizontal band in the central portion of the iris; Ferrão et al., 2016), and *Scinax* sp. 6 (red horizontal band in the central portion of the iris; Ferrão et al., 2016).

*Holotype description*. Adult male (Figs. 2 and 3). Body moderately slender; head wider than body; HL 37% of the SVL; HW 34% of the SVL; head slightly longer than wide (HL/HW = 1.09); snout truncate in dorsal view and rounded in lateral view; END 95% of ED; nostrils slightly protuberant in dorsolateral position; internarial region slightly concave; canthus rostralis rounded; loreal area slightly concave; ED 32% of HL; supratympanic fold weakly marked; TD 48% of ED; vocal sac subgular, bilobate, moderately sized, reaching the pectoral region; vocal slits present, and positioned parallel to edge of the lower lips, between the lateral base of tongue and the angle of jaws; tongue lanceolate; dentigerous processes of vomers triangular, slightly separated, with 4/2 teeth (left/right); choanae oval; pectoral fold present but weakly marked; axillar membrane absent; arm slender; forearm moderately robust; ulnar tubercles indistinct; fingers moderately long and slender, basally webbed, bearing medium-sized elliptical discs (4FD/TD = 0.59); relative length of fingers II<III<V<IV (Fig. 3A); palmar tubercle flat and bifid; thenar tubercle elliptical and moderately protuberant; subarticular tubercle on Finger II subconical; subarticular tubercles on Finger III, IV and V rounded and protuberant; supernumerary tubercles indistinct; nuptial pad white, barely evident covering the preaxial surface of Metacarpal II, not obscuring the thenar tubercle (Fig. 3A); TL 50% of the SVL, THL 47% of the SVL; tarsal fold absent; tarsal tubercles indistinct; FL 37% of the SVL; TAL 75% of FL; internal metatarsal tubercle low and elliptical; external metatarsal tubercle small, rounded, slightly pronounced; subarticular tubercles rounded and pronounced; supernumerary tubercles indistinct; toe discs elliptical (4TD/TD = 0.59); relative toe length I<II<III<V<IV; toe webbing formula I vestigial II 1^+^–2^+^III 1–2^+^ IV 2^+^–1 V (Fig. 3B); cloacal opening directed posteroventrally at the thigh midlevel; skin on dorsum smooth with small tubercles in postocular and scapular regions; vocal sac smooth; chest, belly and ventral surface of the thigh areolate; perianal region with small round warts.

*Holotype measurements (mm)*. SVL 25.9; HL 9.6; HW 8.8; ED 3.2; TD 1.5; UEW 2.4; IND 2.0; TAL 7.2; FL 9.7; HAL 7.6; 3FD 0.9; 4TD 0.9; END 2.9; TL 13.0; THL 12.1; NSD 0.8.

*Holotype colouration*. In preservative, dorsum light grey dorsum (Fig. 2A); anterior portion of the head amber-yellow; a dark brown blotch similar in shape to the moth *Copiopteryx semiramis* extending from the interocular region to middle flanks; infraocular and infratympanic regions white; grey chevron-shaped blotch and dark grey dots on the sacral region; background of hand beige-coloured , arm and forearm with diffuse amber-yellow pigmentation; a narrow horizontal amber-yellow stripe between the hand and forearm and on the anterior portion of the arm; anterior and posterior area of thigh light brown, dorsal surface of thigh beige; beige blotch on the knee; tibia light grey with three diffuse brown stripes; beige-coloured blotch on the tibia-tarsus articulation; tarsus, feet and toes light cream; throat cream with inconspicuous light grey blotches (Fig. 2B); vocal sac and chest cream; belly yellowish cream; white band between the flanks and belly with few light grey blotches; perianal region white. Except by the colour of the bicolored iris (reddish upper and grey lower), the colour in life was not recorded.

*Variation*. The adult paratopotypes and paratypes are similar to the holotype. Variation occurs in the presence and number of small dorsal tubercles (completely absent in some individuals). Variation in foot webbing is as follows: I vestigial II (1–1^1∕2^)–(2–2^1∕2^) III (1–1^1∕3^)–(2–2^1∕2^) IV (2–2^1∕3^)–(1–1^+^) V. Measurements are provided in Appendix S3 and summarized in Table 1.

**Table 1:** Measurements (in mm) of type series of *Scinax ruberoculatus* sp. nov. Values are presented as the mean ± standard deviation, with the range in parentheses.

Measurements	Males (*n* = 28)	Females (*n* = 6)
SVL	24.4 ± 0.87 (22.6–25.9)	26.3 ± 0.73 (25.4–27.5)
HL	9 ± 0.41 (8.3–9.8)	9.6 ± 0.16 (9.4–9.8)
HW	8.4 ± 0.28 (7.9–8.9)	9.1 ± 0.40 (8.6–9.6)
ED	3 ± 0.20 (2.7–3.4)	3 ± 0.15 (2.9–3.2)
TD	1.4 ± 0.12 (1.1–1.6)	1.5 ± 0.07 (1.4–1.6)
UEW	2.3 ± 0.20 (1.8–2.6)	2.4 ± 0.22 (2.1–2.7)
IOD	2.4 ± 0.12 (2.2–2.7)	2.5 ± 0.15 (2.4–2.8)
IND	1.8 ± 0.10 (1.5–2)	1.9 ± 0.12 (1.8–2.1)
TAL	6.6 ± 0.23 (6–7.2)	7.2 ± 0.34 (6.8–7.9)
FL	9.6 ± 0.44 (8.6–10.3)	10.3 ± 0.45 (9.5–10.8)
HAL	6.5 ± 0.35 (5.9–7.6)	7.2 ± 0.53 (6.2–7.8)
3FD	0.9 ± 0.13 (0.7–1.2)	1 ± 0.14 (0.9–1.2)
4TD	1 ± 0.14 (0.7–1.3)	1 ± 0.10 (0.8–1.1)
END	2.8 ± 0.17 (2.4–3.1)	3.1 ± 0.15 (2.9–3.3)
TL	12.3 ± 0.49 (11.2–13.1)	13.4 ± 0.49 (12.9–14.2)
THL	11.5 ± 0.48 (10.2–12.1)	12.2 ± 1.01 (10.2–13.4)

**Notes.**

*n* number of measured specimens

**Figure 4:**
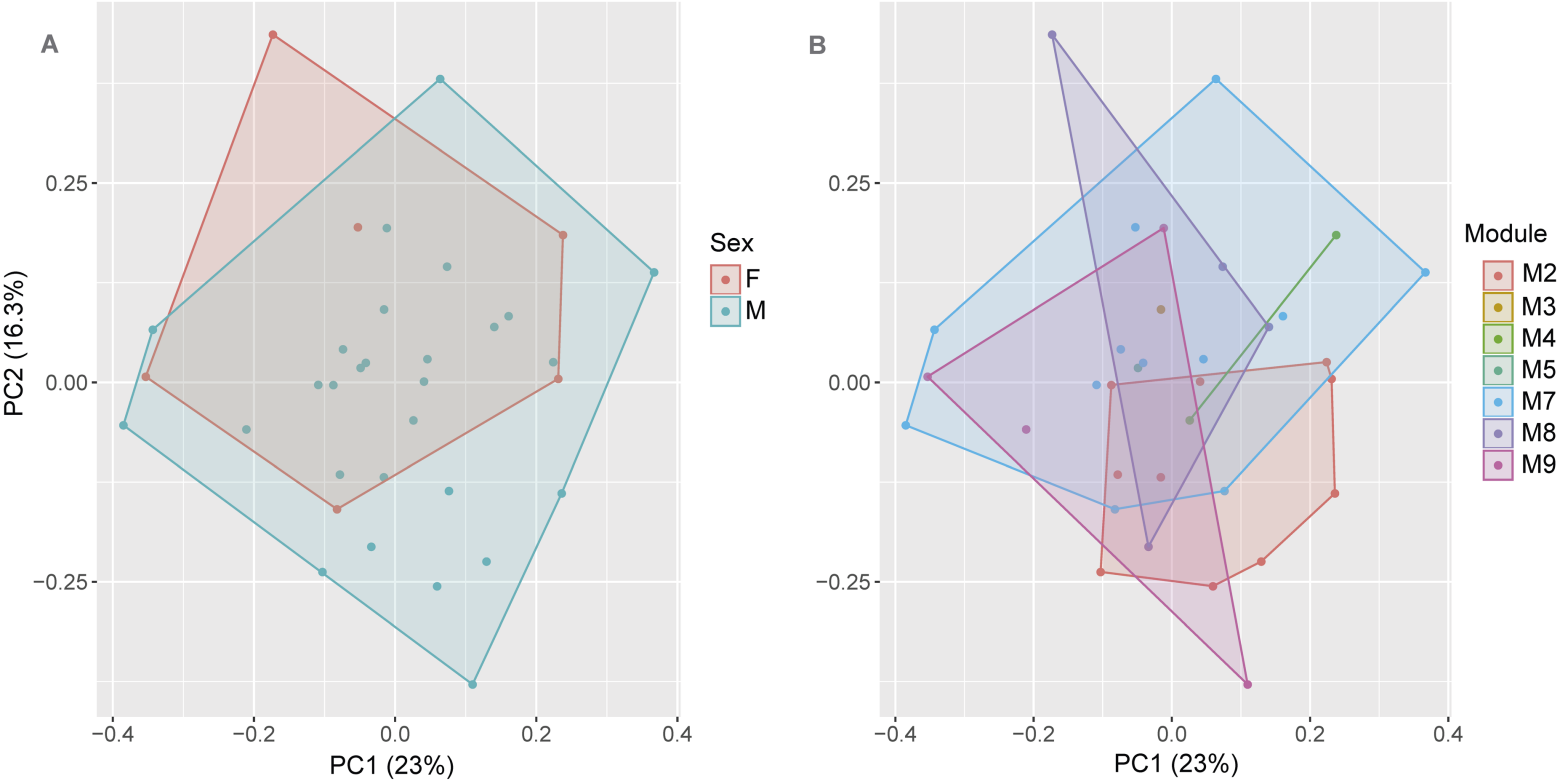
Multivariate morphometric space of *Scinax ruberoculatus* sp. nov. Principal Components Analysis of the SVL and 15 body ratios of males (*N* = 28) and females (*N* = 6). (A) Sampled by sex and (B) sampled by RAPELD sampling modules. Abbreviations: M2–5 and M7–9, RAPELD sampling modules where specimens were collected; F, females; M, males.

The first axis (PC) of the PCA summarized 23% of the variation in the morphometric data, whereas PC2 summarized 16.3%. See Appendix S4 for values of other axes. Sexual dimorphism was not observed in the body shape (morphometric ratios) of *S. ruberoculatus* sp. nov. (Pillai trace = 0.100, *df* = 31, *P* = 0.19), and the body shape of both sexes overlapped in the morphometric multivariate space (Fig. 4A). Males and females of *S. ruberoculatus* sp. nov. did not show distinct morphological variations along the RAPELD sampling modules (Pillai trace = 0.38, *df* = 54, *P* = 0.41). The body shape of specimens from all modules overlapped in the morphometric multivariate space (Fig. 4B).

**Figure 5:**
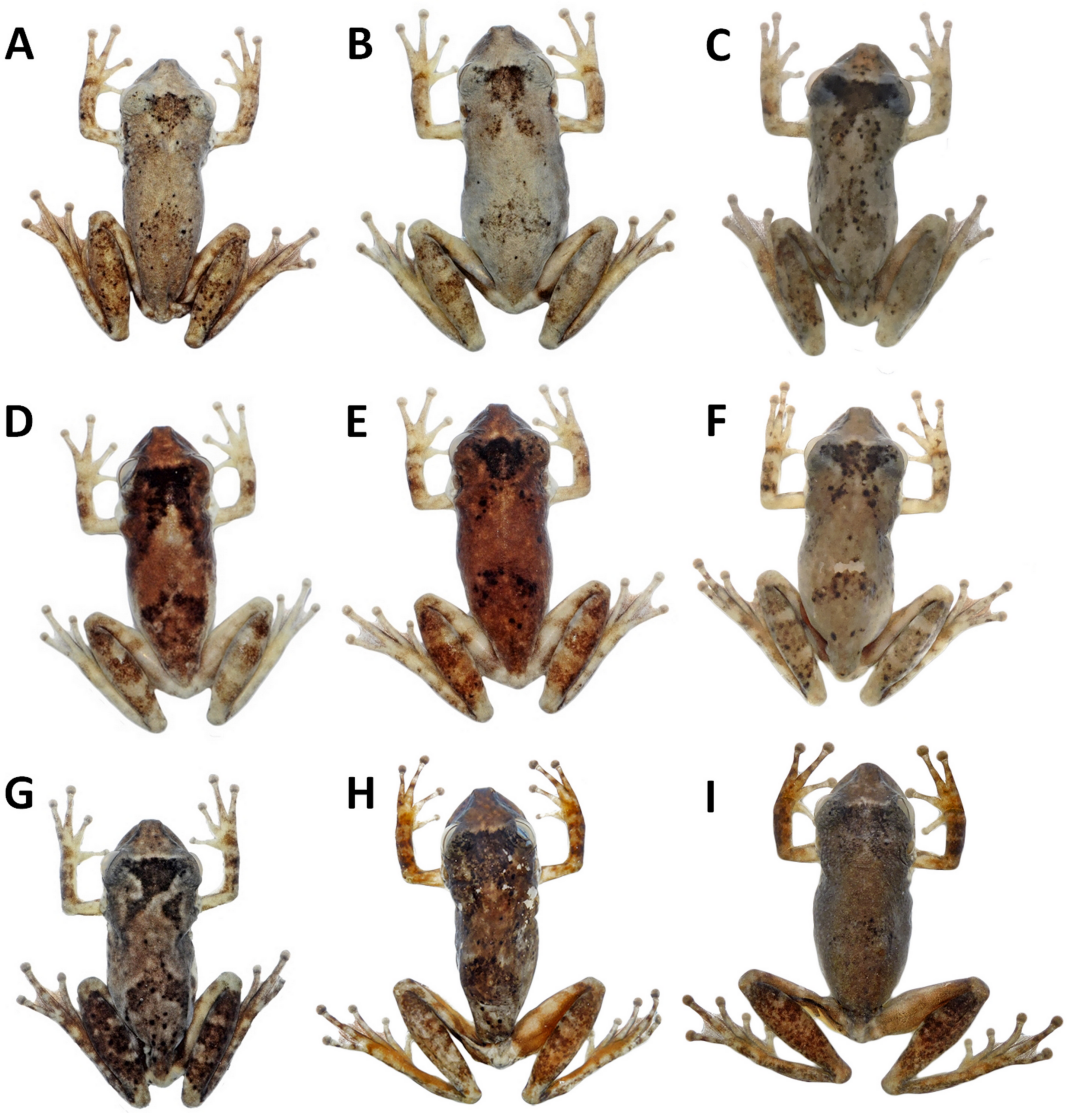
Variation in the dorsal colouration from preserved specimens of *Scinax ruberoculatus* sp. nov. (A) INPA-H 34601, male, SVL 24.1 mm. (B) INPA-H 34600, female, SVL 27.5 mm. (C) INPA-H 34609, male, SVL 24.5 mm. (D) INPA-H 34598, male, SVL 25.5 mm. (E) INPA-H 34614, male, SVL 25.5. (F) INPA-H 34611, male, SVL 23.8 mm. (G) INPA-H 34612, male, SVL 24.4 mm. (H) INPA-H 34618, male, SVL 25.2 mm. (I) INPA-H 34605, female, SVL 26.2 mm. Photos: M Ferrão.

In preservative (Figs. 5A–5I), the colour pattern of the paratypes shows the following variations (frequency in % of paratype specimens): Dorsal ground colour varies from light grey (Fig. 5B) to brown (Fig. 5E). A grey to dark brown blotche on the head and scapular region is shaped like the Neotropical moth *Copiopteryx semiramis* (60%), a human molar in lateral view (26%), or a triangle (14%). A grey to dark brown chevron in the sacral region is present in 42% of paratypes (Figs. 5C–5H). Some individuals have tiny black spots (Figs. 5A–5C) on the dorsal surface of the body and limbs (42%). Light brown or dark grey spots on the upper lip are more concentrated and conspicuous in some individuals (60%). Grey to brown stripe between the eye and the tympanum is present in some individuals (31%); a grey or dark brown supratympanic stripe (95%); a cream to yellowish cream stripe in the lower portion of the flanks bordered by brown spots dorsally (100%). One to three light brown stripes on the dorsal side of the thigh (48%); three grey or brown stripes on the upper tibia (92%); light cream blotch on the heel (100%).Throat, vocal sac, chest and belly vary from cream to yellowish cream; light to dark brown or light to dark grey blotches on the lateral portion of the belly in all paratypes.

**Figure 6:**
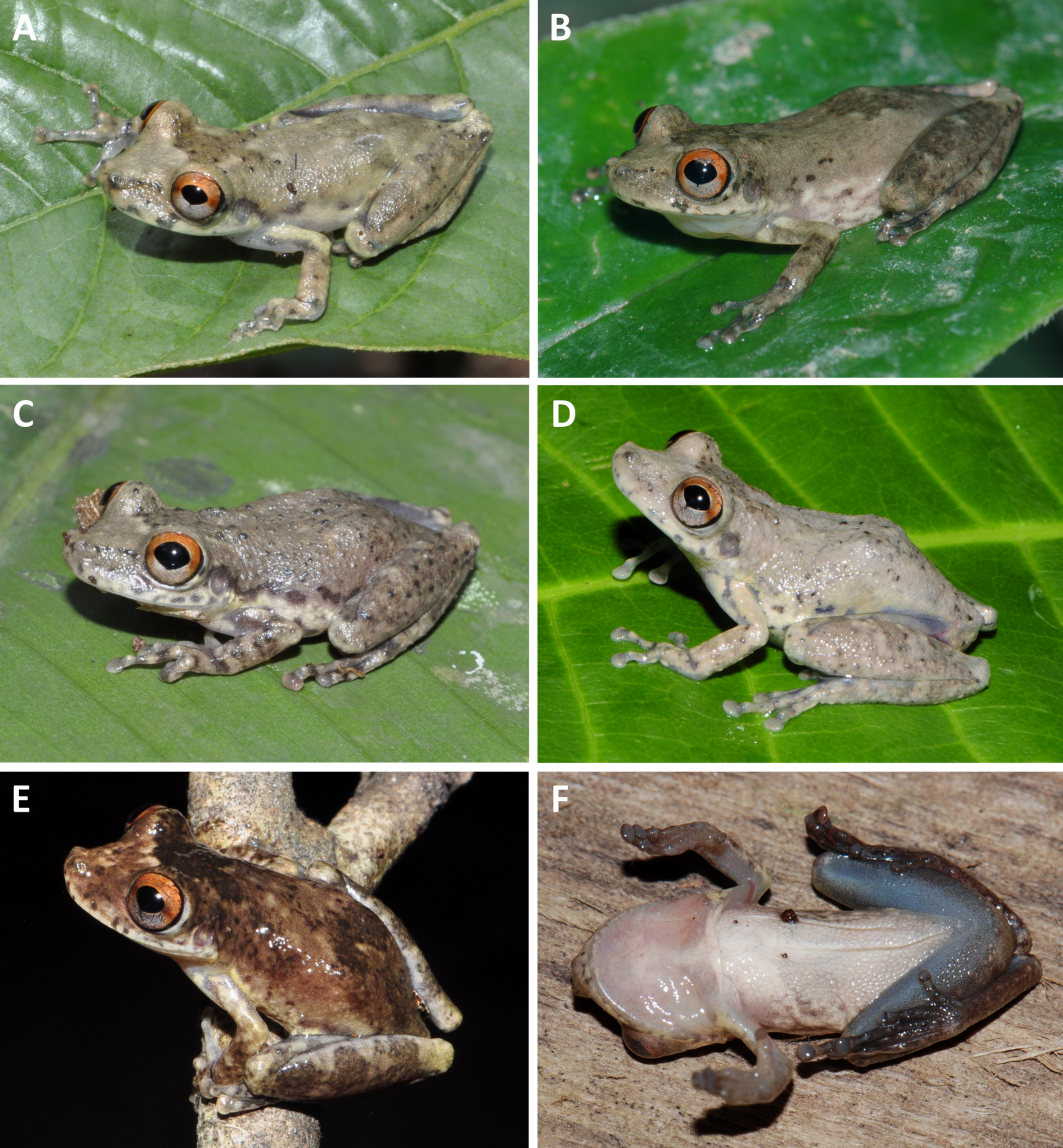
Variation in the colouration of living specimens of the paratypes of *Scinax ruberoculatus* sp. nov. (A) INPA-H 34607, female, SVL 25.4 mm. (B) INPA-H 34602, male, SVL 22.6 mm. (C) INPA-H 34603, male, SVL 23.3 mm. (D) INPA-H 34604, male, SVL 25.8 mm. (E) INPA-H 34623, male, SVL 23.9 mm. (F) INPA-H 34602, male, SVL 22.6 mm. Photos: AP Lima (B, F) and R Fraga (A, C, D, E).

In life (Figs. 6A–6F), dorsal ground colour varies from light grey (Fig. 6D) to brown (Fig. 6E). Blotches, chevrons, or stripes on the dorsal surfaces are more conspicuous than in preservative. Iris bicolored, upper half reddish, lower half grey; both parts separated by a narrow central red streak (Fig. 6). Dorsal surface of the arm cream (Fig. 6A) to yellowish cream (Fig. 6E). Anterior and posterior surfaces of thighs uniformly brown, dorsal surface of thigh yellowish cream to light brown. A whitish cream stripe in the lower portion of the flanks. Throat, vocal sac, and chest grey to semi-translucent white; belly white to greyish-white with light brown to brown blotches laterally (Fig. 6F). Ventral surface of the hand light grey, ventral surface of foot light grey to brown. Webbing light to dark grey.

**Figure 7:**
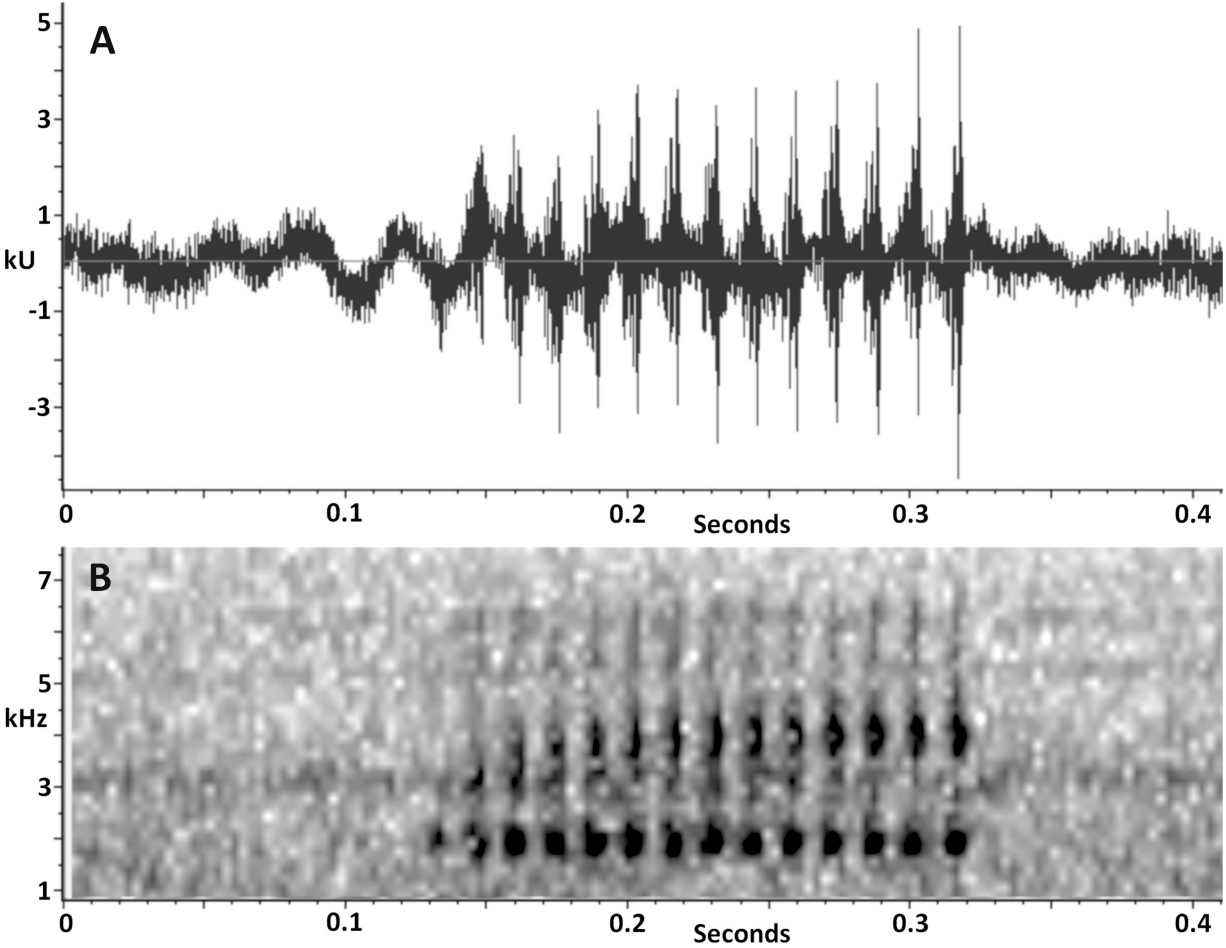
Advertisement call of *Scinax. ruberoculatus* sp. nov. (A) Waveform. (B) Audiospectrogram. Call recorded at RAPELD sampling module 9, Nascentes do Lago Jari National Park, Beruri Municipality, Amazonas, Brazil. Male recorded: INPA-H 34665 (SVL 25.9 mm). Temperature: 25 °C.

*Vocalization*. The advertisement call of *Scinax ruberoculatus* sp. nov. consists of a single moderately long pulsed note (Fig. 7). The quantitative call parameters are as follows (range followed by mean ± standard deviation in parentheses): call duration, 0.134–0.331 s (0.20 ± 0.05, *n* = 20); pulses per call, 10–23 (14.5 ± 3.4, *n* = 20); pulse duration, 0.007–0.009 s (0.008 ± 0.001, *n* = 20); inter-pulse interval, 0.007–0.009 s (0.007 ± 0.001, *n* = 20); pulse repetition rate, 59–71 pulses per sec (66.6 ± 3.6, *n* = 20); dominant frequency, 1,809–1,895 Hz (1,852 ± 19.7, *n* = 20); minimum frequency, 1,170–1,681 Hz (1,452 ± 185, *n* = 20); maximum frequency, 2,378–2,579 Hz (2,420 ± 49, *n* = 20).

**Figure 8:**
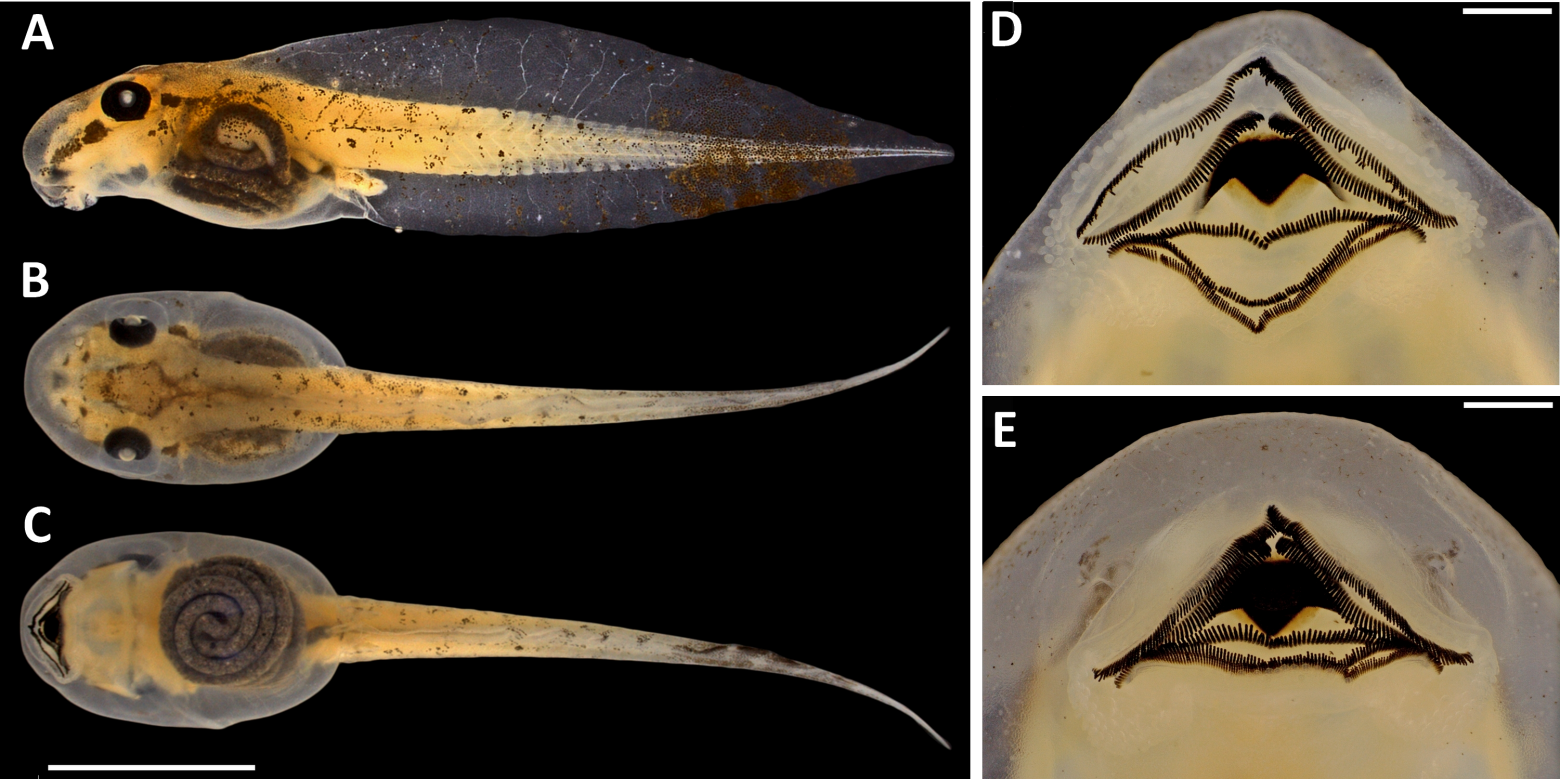
Tadpole of *Scinax. ruberoculatus* sp. nov. (INPA-H 35410) in developmental stage 34. (A) Lateral view. (B) Dorsal view. (C) Ventral views. (D) Details of the opened oral disc. (E) closed oral disc. Tadpoles were collected at RAPELD sampling module 9, Nascentes do Lago Jari National Park, Amazonas, Brazil. Scales for A–C = 5 mm. Scales for D–E = 0.5 mm. Photos: M Ferrão.

*Tadpoles*. The following description is based on one tadpole of the lot INPA-H 35410 in Gosner stage 34 (Fig. 8; Table 2). Body ovoid in dorsal view and almost triangular in lateral view; total length 22.2 mm; body wider than high at the spiracle level (BW/BH = 1.13); snout rounded in dorsal and lateral views; eyes moderately large (ED = 1.0 mm), oriented dorsolaterally and separated by approximately twice the eye diameter (IOD/ED = 1.8); sinistral spiracle; spiracle opening oriented postero-laterally below the midline of the body on the third portion of the body; dextral vent tube, positioned above the margin of the lower fin; vent tube opening on the right side of the ventral fin; slender caudal musculature with greater height than width (TMH/TMW = 1.2) that gradually tapers towards the tail tip; caudal musculature approximately 1.2 higher than the ventral fin and represents 75% of the dorsal fin at the central portion of the tail; dorsal fin originating at the level of the spiracle opening; maximum height of the tail higher than body height (MTH/BH = 1.2). The intestinal mass is visible and positioned subparallel to the longitudinal body axis.

**Table 2:** Morphometric data (in mm) from tadpoles of *Scinax ruberoculatus* sp. nov. Values are presented as the mean ± standard deviation, with the range in parentheses.

Measurements (mm)	Stage 34 (*n* = 3)	Stages 35–38 (*n* = 7)	Stage 39 (*n* = 3)
TL	20.9 ± 1.4 (19.4–22.2)	24.7 ± 1.9 (22.9–27.0)	26.6 ± 1.0 (25.4–27.2)
BL	7.1 ± 0.1 (7.0–7.2)	8.1 ± 0.4 (7.2–8.5)	8.5 ± 0.2 (8.3–8.6)
TAL	13.8 ± 1.3 (12.4–15.0)	16.5 ± 1.3 (14.9–18.5)	18.1 ± 1.1 (16.9–18.9)
BW	4.2 ± 0.3 (3.8–4.4)	5.0 ± 0.3 (4.6–5.4)	5.5 ± 0.1 (5.4–5.5)
BH	3.9 ± 0.4 (3.5–4.3)	4.3 ± 0.2 (4.0–4.7)	4.6 ± 0.1 (4.5–4.6)
TMW	1.5 ± 0.1 (1.4–1.6)	1.9 ± 0.2 (1.6–2.2)	2.2 ± 0.1 (2.1–2.2)
MTH	4.8 ± 0.5 (4.3–5.3)	5.8 ± 0.6 (5.0–6.7)	6.2 ± 0.3 (6.0–6.5)
TMH	1.7 ± 0.1 (1.7–1.8)	2.0 ± 0.1 (1.8–2.1)	2.2 ± 0.1 (2.1–2.2)
IOD	1.8 ± 0.1 (1.8–1.9)	2.1 ± 0.1 (2.0–2.2)	2.3 ± 0.1 (2.3–2.4)
NP	0.6 ± 0.1 (0.6–0.7)	0.8 ± 0.1 (0.6–0.9)	0.9 ± 0.1 (0.8–1.0)
RN	1.4 ± 0.1 (1.3–1.4)	1.5 ± 0.0 (1.5–1.6)	1.6 ± 0.1 (1.5–1.7)
ED	1.1 ± 0.1 (1.0–1.1)	1.1 ± 0.3 (0.7–1.4)	1.3 ± 0.1 (1.2–1.4)
VL	0.7 ± 0.1 (0.6–0.7)	1.0 ± 0.1 (0.7–1.4)	1.1 ± 0.1 (1.0–1.2)
SL	0.7 ± 0.1 (0.6–0.8)	0.9 ± 0.1 (0.7–1.1)	0.8 ± 0.2 (0.6–0.9)

**Notes.**

*n* number of measured tadpoles

Oral disc is in anteroventral position (Fig. 8). Marginal lip papillae bordering the entire posterior region of the lip up to second third of the anterior lip; one row of papillae on the anterior lip and three rows of papillae on the posterior lip. Labial teeth formula 2 (2)/3; A-2 hiatus 0.1 mm; teeth more developed in rows A-1, A-2 and P-1 than in P-2 and P-3. Posterior border of the upper jaw bow with serrated cutting edge; and V-shaped lower jaw with serrated cutting edge.

*Colouration of tadpoles* (stages 34–39, *n* = 13). In live and preserved specimens, bronze body and tail muscle; tiny dark brown spots on body and tail that are denser posteriorly and may resemble small bars in the lower portion of the tail; dark brown stripe from snout to eye; dark brown postocular stripe; translucent tail; dark brown blotches conglomerated in the last third of the tail; and dark brown tip of tail (Fig. 8).

**Figure 9:**
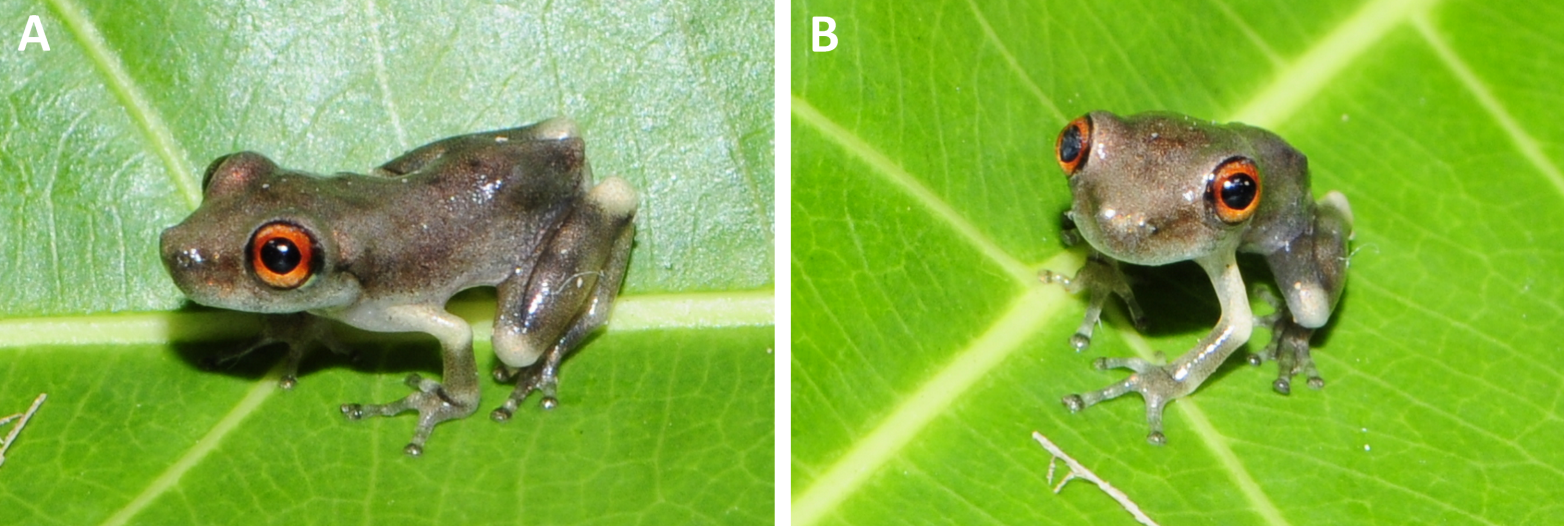
Newly metamorphosed *Scinax. ruberoculatus* sp. nov. (INPA-H 35412). (A) Dorso-lateral view. (B) Fronto-dorsal views. This specimen developed under our care from a typical tadpole of the new species. Photos: R de Fraga.

*Colouration of newly metamorphosed specimens* (INPA-H 35412, *n* = 1). Living specimens, greyish-brown dorsally with small dark grey spots in the sacral region; loreal region brown; iris red with black border; postocular region and anterior portion of flanks brown; inguinal region light grey; dorsal surfaces of arm and elbow, knee and heel cream; three dark grey stripes on dorsal surface of tibia; dorsal surface of discs dark grey; belly light grey, semi-translucent (Fig. 9).

*Notes on the natural history*. Individuals of *Scinax ruberoculatus* sp. nov. were observed mainly in primary and old-growth secondary lowland rainforests (39–68 m a.s.l.) where they occupied edge situations. Its breeding season was correlated with the rainy season in the northern PMRI (November–March). Active males vocalized while sitting on the vegetation in horizontal position 1–2 m above the ground around temporary ponds. The number of calling males was higher on rainy nights. In two large temporary ponds (>25 m^2^) males of *S. ruberoculatus* sp. nov. shared calling sites with *Dendropsophus minutus* (Peters, 1872), *D. rhodopeplus* (Günther, 1858), *D. sarayacuensis* (Shreve, 1935), and *Scinax* sp. 1 (*sensu* Ferrão et al., 2016). Only males of *S. ruberoculatus* sp. nov. were found in small temporary ponds (<4 m^2^). During the day, inactive individuals were observed between leaves of palm trees.

### Species-habitat association

**Figure 10:**
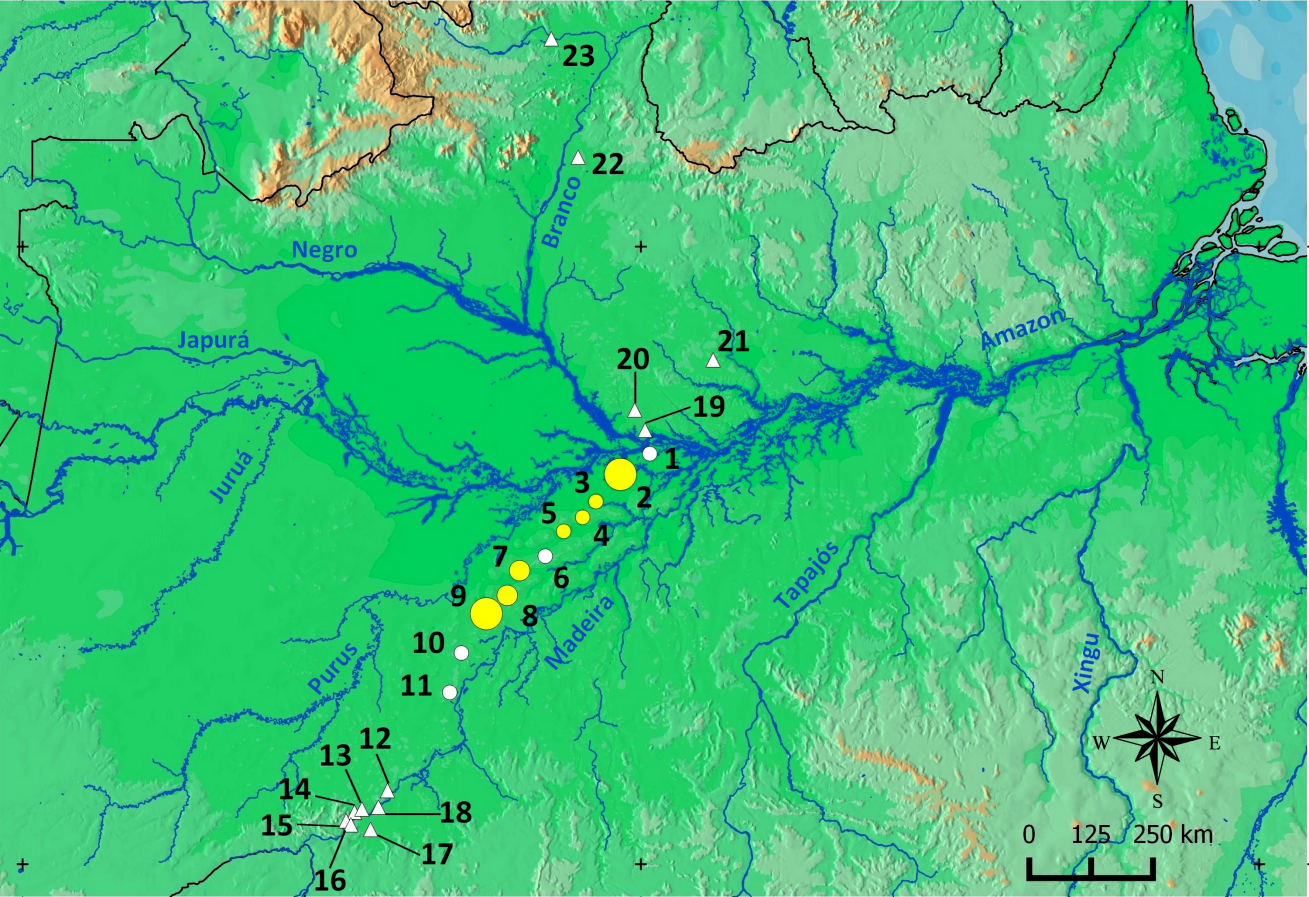
Geographic range of *Scinax. ruberoculatus* sp. nov. Numbers indicate the RAPELD sampling module. Yellow circles: RAPELD sampling modules where the new species was observed. White circles: RAPELD sampling modules where the new species was not observed. Triangles: RAPELD sampling modules outside the study area where the new species was not observed. The diameter of the yellow circles indicates the percentage of plots occupied by the new species within each sampling module (10% in 3–5; 20% in 7–8; 40% in 9; 50% in 2).

**Figure 11:**
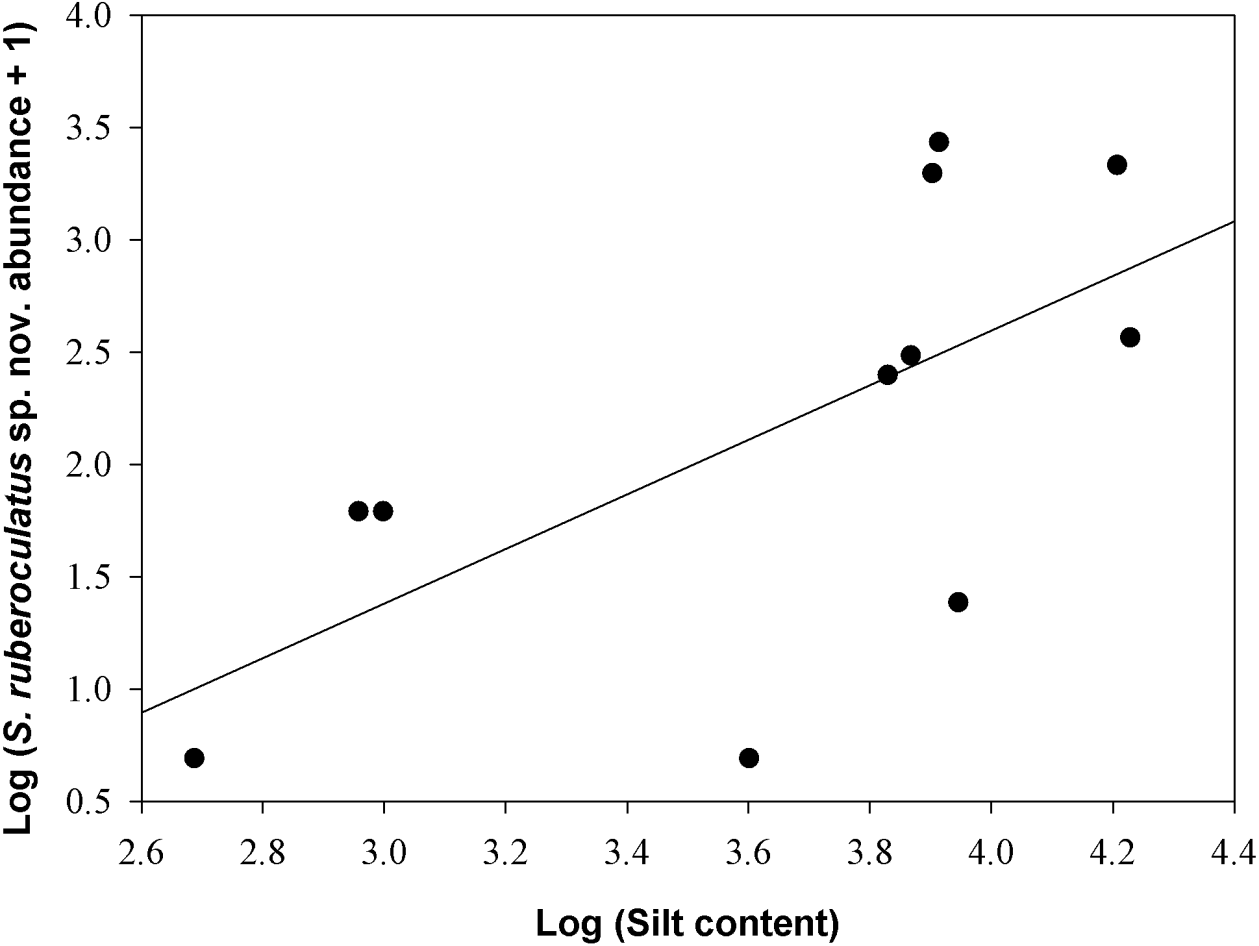
Relationship between the abundance of *Scinax ruberoculatus* sp. nov. and silt content in the Purus-Madeira Rivers Interfluve, Amazonas, Brazil. Model: Log (abundance + 1) = constant + log (silt content). *r*^2^ = 0.41, F1,9 = 6.48, *P* = 0.031.

*Scinax ruberoculatus* sp. nov. is distributed in forested habitat of PMRI along the BR-319 federal highway from RAPELD sampling modules 2 (km 100) to 9 (km 450) (Fig.  10). The zero-inflated model revealed a significative and positive effect of soil silt content on the occurrence of this species across the whole study area (occurrence = −5.836 intercept −4.978 number of trees + 3.402 silt content + 0.242 underground water; Θ = 3.299 silt *P* = 0.012). The soil silt content also explained 41% of the abundance of *S. ruberoculatus* sp. nov. (Fig. 11) in the plots where the species was found (abundance = −2.261 + 1.214 silt content; *r*^2^ = 0.41, F1,9 = 6.48, *P* = 0.031). Forest structure (*P* = 0.567) and underground water (*P* = 0.260) did not have a significant effect on the abundance of the new species.

## Discussion

*Scinax ruberoculatus* sp. nov. is the 72nd described species of the genus *Scinax* (*sensu* Duellman, Marion & Hedges, 2016) and the 30th *Scinax* species known to occur in the Amazonia *sensu lato* (see Table S1). Recent studies have demonstrated that the species richness of the genus *Scinax* is greatly underestimated (Fouquet et al., 2007; Ferrão et al., 2016; Menezes et al., 2016). Despite that, the rate of species descriptions for Amazonian *Scinax* has been low in the last decade (2006–2016; 0.3 species per year) compared with that of other frog genera, e.g., *Allobates* Zimmermann and Zimmermann, 1988 (0.8 species per year) and *Boana* Gray, 1825 (0.7 species per year). However, the description rate of Amazonian *Scinax* may increase in the next years. At least other six potential unnamed species of *Scinax* pending formal description have recently been reported in the PMRI (Ferrão et al., 2016). Additionally, the geographic distribution of some species (e.g., *S. blairi*, *S. cruentomma*, *S. iquitorum*, and *S. wandae*) needs to be reviewed.

Over the past decade, our research group has conducted standardized frog sampling along 23 permanent RAPELD sampling modules and/or grids (∼450 plots) distributed across a longitudinal gradient of approximately 1,500 km in the Brazilian Amazonia (Fig. 10). Despite this high level of sampling effort, *S. ruberoculatus* sp. nov. has only been observed in seven sampling modules in northern PMRI. However, sampling gaps occur in areas closer to the banks of the PMRI and the neighbouring interfluves in the southern Amazon; therefore, the range of *S. ruberoculatus* sp. nov. may be broader than shown here.

In this study, we addressed the influence of environmental heterogeneity on the distribution of a species of the genus *Scinax* in Amazonia for the first time. Our results indicated that the occurrence and abundance of *S. ruberoculatus* sp. nov. is positively affected by the silt content in the soil. An explanation for this phenomenon can be seen in the fact that increased silt content directly reduces the soil drainage capacity (Juo & Franzluebbers, 2003). In areas with slight altitude variations (1–3 m in the northern PMRI; Rossetti, Toledo & Góes, 2005), temporary water bodies in silty soils persist for longer periods of time compared with temporary ponds in well-drained, sandier substrates. Because temporary water bodies play a crucial role in the reproduction of *S. ruberoculatus* sp. nov., the soil structure represents an important ecological factor that affects the spatial distribution of the species in the study area. Alternatively, the soil texture may also affect the species composition of invertebrates in Amazonia, such as ants (Vasconcelos, Macedo & Vilhena, 2003; Oliveira et al., 2009; Souza et al., 2016), mites (Moraes et al., 2011) and termites (Dambros et al., 2013). Therefore, the distribution of *S. ruberoculatus* sp. nov. could be indirectly affected by the soil texture, which promotes the availability of certain invertebrate groups that represent prey species. However, the relationships between invertebrates and soil texture have not been investigated in the PMRI, and additional data are necessary to test this hypothesis.

Environmental heterogeneity may have different effects on frog distribution and abundance in the northern Amazon River depending on the reproductive mode (Menin et al., 2007; Menin, Waldez & Lima, 2011). Species that exhibit terrestrial reproduction are primarily affected by soil characteristics, such as the clay content and pH, whereas species that exhibit an aquatic reproductive mode are influenced by the tree density and distance from streams (Landeiro, Waldez & Menin, 2014). Since *S. ruberoculatus* sp. nov. uses temporary ponds to reproduction, our results are inconsistent with the above generalization made for aquatic breeding frogs but similar with generalization for terrestrial breeding frogs. Nevertheless, we conclude that environmental variables with the ability to filter the occurrence and abundance of species can also cause regional variations in species-habitat associations, independent of the species’ reproductive modes. Future investigations should include additional frog species and a wider spatial scale to further elucidate this relationship.

By investigating a species of snouted treefrog, our study showed that alpha-taxonomy and ecology can be integrated into a single framework via the sampling of standardized units along a heterogeneous Amazonian landscape. Such an approach has the potential to reveal additional species and their ecological relationships not only in the study area but also in other megadiverse regions for which insufficient data on both the biota and environmental predictors are still available.

##  Supplemental Information

10.7717/peerj.4321/supp-1 Table S1 Species of *Scinax* occurring in Amazonia

10.7717/peerj.4321/supp-2 Appendix S1 Specimens examined for morphological comparisons Abbreviations: (AM) Highway at State of Amazonas, Brazil; (PDBFF) Projeto Dinâmica Biológica de Fragmentos Florestais (a project in Brazil focused on dynamics of forest fragments), (km) kilometre; (INPA-H) Herpetological Section of the Zoological Collection of the Instituto Nacional de Pesquisas da Amazônia, Manaus, Brazil; (RMNH) Nationaal Natuurhistorisch Museum, Leiden, The Netherlands; (QCAZ) Museo de Zoología, Pontificia Universidad Católica del Ecuador, Quito, Ecuador; (KU) University of Kansas, Museum of Natural History, Division of Herpetology, Lawrence, Kansas, USA; (ANDES-A) Museo de Historia Natural ANDES, Universidad de los Andes, Bogotá, Colombia; (ZFMK) Zoologisches Forschungsinstitut und Museum Alexander Koenig, Herpetologische Abteilung, Adenauerallee, Germany.

10.7717/peerj.4321/supp-3 Appendix S2 Sampling methods for the environmental variables

10.7717/peerj.4321/supp-4 Appendix S3 Morphometric measurements of *Scinax ruberoculatus* sp. nov. from Purus-Madeira Rivers Interfluve, State of Amazonas, Brazil Abbreviations: (INPA-H) Herpetological Section of the Zoological Collection of the Instituto Nacional de Pesquisas da Amazônia, Manaus, Brazil, (RAPELD) sampling sites, (M) male, (F) female, (SVL) snout-vent length, (HL) head length, (HW) head width, (ED) horizontal eye diameter, (UEW) upper eyelid width, (IND) internarial distance, (IOD) interorbital distance, (TD) horizontal tympanum diameter, (TL) tibia length, (FL) foot length, (END) eye-nostril distance, (3FD) third finger disk diameter, (4TD) fourth toe disk diameter, (TAL) tarsus length, (HAL) hand length, (THL) thigh length.

10.7717/peerj.4321/supp-5 Appendix S4 Principal Components resulting from the Principal Component Analyses conducted for the SVL and 15 morphometric ratios from all individuals of *Scinax ruberoculatus* sp. nov. (males and females) Abbreviations: (PC) Principal Component, (SVL) snout-vent length, (HL) head length, (HW) head width, (ED) horizontal eye diameter, (UEW) upper eyelid width, (IND) internarial distance, (IOD) interorbital distance, (TD) horizontal tympanum diameter, (TL) tibia length, (FL) foot length, (END) eye-nostril distance, (3FD) third finger disk diameter, (4TD) fourth toe disk diameter, (TAL) tarsus length, (HAL) hand length, (THL) length thigh.

## Additional Information and Declarations

### Data Availability

The following information was supplied regarding data availability: The raw data is included in Appendix S3.

### New Species Registration

The following information was supplied regarding the registration of a newly described species: Genus name: urn:lsid:zoobank.org:act:6C6C5E27-39C7-49F4-AE2D-B41DCE6C17C2 New species name: *Scinax ruberoculatus* urn:lsid:zoobank.org:act:2B83C876-453B-4199-A872-5CED04DB25A5 Publication LSID: urn:lsid:zoobank.org:pub:D50A0044-0EA6-4619-B0FF-42A7443C4EAB.

## References

[ref-1] Allmon WD (1991). A plot study of forest floor litter frogs, Central Amazon, Brazil. Journal of Tropical Ecology.

[ref-2] Altig R, McDiarmid RW, McDiarmid RW, Altig R (1999). Body plan: development and morphology. Tadpoles: the biology of anuran larvae.

[ref-3] Araujo-Vieira K, Brandão RA, Faria DCC (2015). A new species of rock-dwelling *Scinax* Wagler (Anura: Hylidae) from Chapada dos Veadeiros, central Brazil. Zootaxa.

[ref-4] Araujo-Vieira K, Valdujo PH, Faivovich J (2016). A new species of *Scinax* Wagler (Anura: Hylidae) from Mato Grosso, Brazil. Zootaxa.

[ref-5] Barrio-Amorós CL, Orellana A, Chacón-Ortiz A (2004). A new species of *Scinax* (Anura: Hylidae) from the Andes of Venezuela. Journal of Herpetology.

[ref-6] Becker CG, Fonseca CR, Haddad CFB, Batista RF, Prado PI (2007). Habitat split and the global decline of amphibians. Science.

[ref-7] Bioacoustics Research Program (2014). http://www.birds.cornell.edu/brp/raven/RavenOverview.html.

[ref-8] Block WM, Brennan LA (1993). The habitat concept in ornithology. Currently Ornithology.

[ref-9] Bokermann WCA (1968). Three new *Hyla* from the Plateau of Maracás, central Bahia, Brazil. Journal of Herpetology.

[ref-10] Bortolus A (2006). The austral cordgrass *Spartina densiflora* Brong: its taxonomy, biogeography and natural history. Journal of Biogeogrphy.

[ref-11] Bortolus A (2008). Error cascades in the biological sciences: the unwanted consequences of using bad taxonomy in ecology. AMBIO.

[ref-12] Bortolus A, Schwindt E, Iribarne O (2002). Positive plant–animal interactions in the high marsh of an Argentinean coastal lagoon. Ecology.

[ref-13] Brusquetti F, Jansen M, Barrio-Amarós C, Segalla M, Haddad CFB (2014). Taxonomic review of *Scinax fuscomarginatus* (Lutz, 1925) and related species (Anura; Hylidae). Zoological Journal of the Linnean Society.

[ref-14] Bueno AS, Bruno RS, Pimentel TP, Sanaiotti TM, Magnusson WE (2012). The width of riparian habitats for understory birds in an Amazonian forest. Ecological Applications.

[ref-15] Caminer MA, Milá B, Jansen M, Fouquet A, Venegas PJ, Chávez G, Lougheed SC, Ron SR (2017). Systematics of the *Dendropsophus leucophyllatus* species complex (Anura: Hylidae): cryptic diversity and the description of two new species. PLOS ONE.

[ref-16] Campbell HW, Christman SP, Scott NJ (1982). Field techniques for herpetofaunal community analysis. Herpetological communities: a symposium of the society for the study of amphibians and reptiles and the herpetologists’ league.

[ref-17] Caramaschi U, Cardoso MCS (2006). Taxonomic status of Hyla camposseabrai Bokermann, 1968 (Anura: Hylidae). Journal of Herpetology.

[ref-18] Cardoso AJ, Sazima I (1980). Nova espécie de *Hyla* do sudeste brasileiro (Amphibia, Anura, Hylidae). Revista Brasileira de Biologia.

[ref-19] Carvalho TR, Teixeira BFV, Duellman WE, Giaretta AA (2015). *Scinax cruentommus* (Anura: Hylidae) in the upper Rio Negro drainage, Amazonas state, Brazil, with the redescription of its advertisement call. Phyllomedusa.

[ref-20] Carvalho-e Silva SP, Peixoto OL (1991). Duas novas espécies de *Ololygon* para os Estados do Rio de Janeiro e Espírito Santo (Amphibia, Anura, Hylidae). Revista Brasileira de Biologia.

[ref-21] Castilho CV, Magnusson WE, Araújo RNO, Luizão RCC, Luizão FJ, Lima AP, Higuchi N (2006). Variation in aboveground tree life biomass in a central Amazonian forest: effects of soil and topography. Forest Ecology and Management.

[ref-22] Cei JM (1980). Amphibians of Argentina. Monitore Zoologico Italiano, Monografia.

[ref-23] Cintra BBL, Schietti J, Emillio T, Martins D, Moulatlet G, Souza P, Levis C, Quesada CA, Schöngart J (2013). Soil physical restrictions and hydrology regulate stand age and wood biomass turnover rates of Purus–Madeira interfluvial wetlands in Amazonia. Biogeosciences.

[ref-24] Conte CE, Araujo-Vieira K, Crivellari LB, Berneck BVM (2016). A new species of *Scinax* Wagler (Anura: Hylidae) from Paraná, southern Brazil. Zootaxa.

[ref-25] Corlett RT, Primack RB (2011). Tropical rain forests: an ecological and biogeographical comparison.

[ref-26] Dambros CS, Silva VNV, Azevedo R, Morais JW (2013). Road-associated edge effects in Amazonia change termite community composition by modifying environmental conditions. Journal for Nature Conservation.

[ref-27] De la Riva I (1993). A new species of *Scinax* (Anura, Hylidae) from Argentina and Bolivia. Journal of Herpetology.

[ref-28] Dexter KG, Pennington TD, Cunningham CW (2010). Using DNA to assess errors in tropical tree identifications: how often are ecologists wrong and when does it matter?. Ecological Monographs.

[ref-29] Dias-Terceiro RG, Kaefer IL, Fraga R, Araújo MC, Simões PI, Lima AP (2015). A matter of scale: historical and environmental factors structure anuran assemblages from the Upper Madeira River, Amazonia. Biotropica.

[ref-30] Drummond LO, Baêta D, Pires MRS (2007). A new species of *Scinax* (Anura, Hylidae) of the S. ruber clade from Minas Gerais, Brazil. Zootaxa.

[ref-31] Duellman WE (1970). The hylid frogs of Middle America 1. Monograph of the Museum of Natural History, the University of Kansas.

[ref-32] Duellman WE (1971). The identities of some Ecuadorian hylid frogs. Herpetologica.

[ref-33] Duellman WE (1972a). South American frogs of the *Hyla rostrata* group (Amphibia, Anura, Hylidae). Zoologische Mededelingen. Leiden.

[ref-34] Duellman WE (1972b). A new species of *Hyla* from Amazonian Ecuador. Copeia.

[ref-35] Duellman WE (1973). Descriptions of new hylid frogs from Colombia and Ecuador. Herpetologica.

[ref-36] Duellman WE (1978). The biology of an equatorial herpetofauna in Amazonian Ecuador. Miscellaneous Publications of the University of Kansas Museum of Natural History.

[ref-37] Duellman WE (1986). Two new species of *Ololygon* (Anura: Hylidae) from the Venezuelan Guyana. Copeia.

[ref-38] Duellman WE (2005). Cusco Amazónico: the lives of amphibians and reptiles in an Amazonian rainforest.

[ref-39] Duellman WE, Marion AB, Hedges SB (2016). Phylogenetics, classification, and biogeography of the treefrogs (Amphibia: Anura: Arboranae). Zootaxa.

[ref-40] Duellman WE, Wiens JJ (1992). The status of the hylid frog genus *Ololygon* and the recognition of *Scinax* Wagler, 1830. Occasional Papers of the Museum of Natural History, University of Kansas.

[ref-41] Duellman WE, Wiens JJ (1993). Hylid frogs of the genus *Scinax* Wagler, 1830, in Amazonian Ecuador and Peru. Occasional Paper of the Museum of Natural History, University of Kansas.

[ref-42] Eva HD, Huber O (2005). A proposal for defining the geographical boundaries of Amazonia. Rep. EUR 21808-EN.

[ref-43] Fabrezi M, Alberch P (1996). The carpal elements of anurans. Herpetologica.

[ref-44] Faivovich J (2002). A cladistic analysis of *Scinax* (Anura: Hylidae). Cladistics.

[ref-45] Faivovich J, Haddad CFB, Garcia PCA, Frost DR, Campbell JA, Weeler WC (2005). Systematic review of the frog family Hylidae, with special reference to Hylinae: phylogenetic analysis and taxonomic revision. Bulletin of the American Museum of Natural History.

[ref-46] Ferrão M, Colatreli O, Fraga R, Kaefer IL, Moravec J, Lima AP (2016). High species richness of *Scinax* treefrogs (Hylidae) in a threatened Amazonian Landscape revealed by an integrative approach. PLOS ONE.

[ref-47] Ferrão M, Moravec J, Fraga R, Almeida AP, Kaefer IL, Lima AP (2017). A new species of *Scinax* from the Purus-Madeira interfluve, Brazilian Amazonia (Anura, Hylidae). ZooKeys.

[ref-48] Fouquet A, Vences M, Salducci MD, Meyer A, Marty C, Gilles A (2007). Revealing cryptic diversity using molecular phylogenetics and phylogeography in frogs of the *Scinax ruber* and *Rhinella margaritifera* species groups. Molecular Phylogenetics and Evolution.

[ref-49] Fouquette MJ, Pyburn WF (1972). A new Colombian treefrog of the *Hyla rubra* complex. Herpetologica.

[ref-50] Frost DR (2017). http://research.amnh.org/herpetology/amphibia/index.html.

[ref-51] Giam X, Scheffers BR, Sodhi NS, Wilcove DS, Ceballos G, Ehrlich PR (2012). Reservoirs of richness: least disturbed tropical forest are centres of undescribed species diversity. Proceedings of the Royal Society.

[ref-52] Gomes MR, Alves ACR, Peixoto O (2014). O girino de *Scinax nebulosus* (Amphibia, Anura, Hylidae). Iheringia: Série Zoologia.

[ref-53] Gosner KL (1960). A simplified table for staging anuran embryos and larvae with notes on identification. Herpetologica.

[ref-54] Gotelli NJ (2004). A taxonomic wish-list for community ecology. Philosophical Transactions of the Royal Society B: Biological Science.

[ref-55] Hero JM, Mijares-Urrutia A (1995). The tadpole of Scinax rostrata. Journal of Herpetology.

[ref-56] Heyer WR, Rand AS, Cruz CAG, Peixoto OL, Nelson CE (1990). Frogs of Boracéia. Arquivos de Zoologia.

[IBGE(1997] IBGE–Instituto Brasileiro de Geografia e Estatística (1997). Recursos naturais e meio ambiente: uma visão do Brasil.

[ref-58] Isaac NJB, Mallet J, Mace GM (2004). Taxonomic inflation: its influence on macroecology and conservation. Trends in Ecology & Evolution.

[ref-59] Jackman S (2015). http://pscl.stanford.edu/.

[ref-60] Jenkins CN, Pimm SL, Joppa LN (2013). Global patterns of terrestrial vertebrate diversity and conservation. Proceedings of the National Academy of Sciences of the United States of America.

[ref-61] Jorge RF, Simões PI, Magnusson WE, Lima AP (2016). Fine-scale habitat heterogeneity explains the local distribution of two Amazonian frog species of concern for conservation. Biotropica.

[ref-62] Juncá FA, Napoli MF, Nunes I, Mercês EA, Abreu RO (2015). A new species of the *Scinax ruber* clade (Anura, Hylidae) from the Espinhaço Range, northeastern Brazil. Herpetologica.

[ref-63] Jungfer K-H, Faivovich J, Padial JM, Castroviejo-Fisher S, Lyra MM, Berneck B, Iglesias PP, Kok PJR, MacCulloch RD, Rodrigues MT, Verdade VK, Torres Gastello CP, Chaparro JC, Valdujo PH, Reichle S, Moravec J, Gvoždík V, Gagliardi-Urrutia G, Ernst R, De la Riva I, Means DB, Lima AP, Señaris JC, Wheeler WC, Haddad CFB (2013). Systematics of spiny-backed treefrogs (Hylidae: *Osteocephalus*): an Amazonian puzzle. Zoologica Scripta.

[ref-64] Juo ASR, Franzluebbers K (2003). Tropical soils: properties and management for sustainable agriculture.

[ref-65] Kats LB, Ferrer RP (2003). Alien predators and amphibian declines: review of two decades of science and the transition to conservation. Diversity and Distributions.

[ref-66] Köhler J, Jansen M, Rodriguez A, Kok PJR, Toledo LP, Emmrich M, Glaw F, Haddad CFB, Rödel MO, Vences M (2017). The use of bioacoustics in anuran taxonomy: theory, terminology, methods and recommendations for best practice. Zootaxa.

[ref-67] Kreft H, Jetz W (2007). Global patterns and determinants of vascular plant diversity. Proceedings of the National Academy of Sciences of the United States of America.

[ref-68] Landeiro VL, Waldez F, Menin M (2014). Spatial and environmental patterns of Amazonian anurans: differences between assemblages with aquatic and terrestrial reproduction, and implications for conservation management. Natureza & Conservação.

[ref-69] Lescure J, Marty C (2000). Atlas des Amphibiens de Guyane. Collections Patrimoines Naturels.

[ref-70] Lima LP, Bastos RP, Giaretta AA (2005). A new *Scinax* Wagler, 1830 of the *S. rostratus* group from central Brazil (Amphibia, Anura, Hylidae). Arquivos do Museu Nacional. Rio de Janeiro.

[ref-71] Limbaugh BA, Volpe EP (1957). Early development of the Gulf Coast toad, *Bufo valliceps* Weigmann. American Museum Novitates.

[ref-72] Lips KR, Brem F, Brenes R, Reeve JD, Alford RA, Voyles J, Carey C, Livo L, Pessier AP, Collins JP (2006). Emerging infectious disease and the loss of biodiversity in a Neotropical amphibian community. Proceedings of the National Academy of Sciences of the United States of America.

[ref-73] Lutz B (1973). Brazilian species of Hyla.

[ref-74] Magnusson WE, Braga-Neto R, Pezzini F, Baccaro F, Bergallo H, Penha J, Rodrigues D, Lima AP, Albernaz A, Hero JM, Lawson B, Castilho C, Drucker C, Franklin E, Mendonça F, Costa F, Galdino G, Castley G, Zuanon J, Vale J, Santos JLC, Luizão R, Cintra R, Barcosa RI, Lisboa A, Koblitz RV, Cunha CN, Pontes ARM (2013). Biodiversidade e monitoramento ambiental integrado: o sistema RAPELD na Amazônia.

[ref-75] Magrini L, Carvalho-e Silva SP, Béda AF, Giaretta AA (2011). Calls of five species of the *Scinax ruber* (Anura: Hylidae) clade from Brazil with comments on their taxonomy. Zootaxa.

[ref-76] Maldonado FD, Keizer EWH, Graça PMLA, Fearnside PM, Vitel CS, Sousa-Junior WC, Waichman AV, Sinisgalli PAA, Angelis CF, Romeiro AR (2012). Previsão temporal da distribuição espacial do desmatamento no interflúvio Purus-Madeira até o ano 2050. Rio Purus: Água, Território e Sociedade na Amazônia Sul-Ocidental.

[ref-77] Martins DL, Schietti J, Feldpausch TR, Luizão FJ, Oliver L, Andrade A, Castilho CV, Laurance SG, Oliveira Á, Ieda L, Quesada CA (2014). Soil-induced impacts on forest structure drive coarse woody debris stocks across central Amazonia. Plant Ecology & Diversity.

[ref-78] McDiarmid RW, Altig R (1990). Description of a bufonid and two hylid tadpoles from western Ecuador. Alytes.

[ref-79] Menezes L, Canedo C, Batalha-Filho H, Garda AA, Gehara M, Napoli MF (2016). Multilocus Phylogeography of the Treefrog *Scinax eurydice* (Anura, Hylidae) reveals a plio-pleistocene diversification in the Atlantic Forest. PLOS ONE.

[ref-80] Menin M, Lima AP, Magnusson WE, Waldez F (2007). Topographic and edaphic effects on the distribution of terrestrially reproducing anurans in Central Amazonia: mesoscale spatial patterns. Journal of Tropical Ecology.

[ref-81] Menin M, Waldez F, Lima AP (2011). Effects of environmental and spatial factors on the distribution of anuran species with aquatic reproduction in Central Amazonia. Herpetological Journal.

[ref-82] Moraes J, Franklin E, Morais JW, Souza JLP (2011). Species diversity of edaphic mites (Acari: Oribatida) and effects of topography, soil properties and litter gradients on their qualitative and quantitative composition in 64 km2 of forest in Amazonia. Experimental and Applied Acarology.

[ref-83] Moravec J, Tuanama IA, Pérez-Peña PE, Lehr E (2009). A new species of *Scinax* (Anura: Hylidae) from the area of Iquitos, Amazonian Peru. South American Journal of Herpetology.

[ref-84] Myers CW, Duellman WE (1982). A new species of *Hyla* from Cerro Colorado, and other tree frog records and geographical notes from Western Panama. American Museum Novitates.

[ref-85] Napoli MF (2005). A new species of allied to *Hyla circumdata* (Anura: Hylidae) from Serra da Mantiqueira, southeastern Brazil. Herpetologica.

[ref-86] Nunes I, Carvalho Jr RR, Pereira EG (2010). A new species of *Scinax* Wagler (Anura: Hylidae) from Cerrado of Brazil. Zootaxa.

[ref-87] Nunes I, Kwet A, Pombal Jr JP (2012). Taxonomic revision of the *Scinax alter* species complex (Anura: Hylidae). Copeia.

[ref-88] Nunes I, Pombal Jr JP (2010). A new *Scinax* Wagler (Amphibia, Anura, Hylidae) from the Atlantic rain forest remains of southern State of Bahia, north-eastern Brazil. Amphibia-Reptilia.

[ref-89] Nunes I, Pombal Jr JP (2011). A new snouted treefrog of the speciose genus *Scinax* Wagler (Anura, Hylidae) from northeastern Brazil. Herpetologica.

[ref-90] Oliveira PY, Souza JLP, Baccaro FB, Franklin E (2009). Ant species distribution along a topographic gradient in a “terra-firme” forest reserve in Central Amazonia. Pesquisa Agropecuária Brasileira.

[ref-91] Pimm SL, Jenkins CN, Abell R, Brooks TM, Gittleman JL, Joppa LN, Raven PH, Roberts CM, Sexton JO (2014). The biodiversity of species and their rates of extinction, distribution, and protection. Science.

[ref-92] Pombal Jr JP, Bastos RP, Haddad CFB (1995). Vocalizações de algumas espécies do gênero *Scinax* (Anura, Hylidae) do sudeste do Brasil e comentários taxonômicos. Naturalia.

[ref-93] Pombal Jr JP, Bilate M, Gambale PG, Signorelli L, Bastos RP (2011). A new miniature treefrog of the *Scinax ruber* clade from the Cerrado of Central Brazil (Anura: Hylidae). Herpetologica.

[ref-94] Pombal Jr JP, Haddad CFB, Kasahara S (1995). A new species of *Scinax* (Anura: Hylidae) from southeastern Brazil, with comments on the genus. Journal of Herpetology.

[ref-95] Pons J, Barraclough TG, Gomez-Zurita J (2006). Sequence based species delimitation for the DNA taxonomy of undescribed insects. Systematic Biology.

[ref-96] Pugliese A, Baêta D, Pombal Jr JP (2009). A new species of *Scinax* (Anura: Hylidae) from rocky montane fields in southeastern and central Brazil. Zootaxa.

[ref-97] Pugliese A, Pombal Jr JP, Sazima I (2004). A new species of *Scinax* (Anura: Hylidae) from rocky montane fields of the Serra do Cipo, southeastern Brazil. Zootaxa.

[ref-98] Puillandre N, Lambert A, Brouillet S, Achaz G (2012). ABGD, Automatic Barcode Gap Discovery for primary species delimitation. Molecular Ecology.

[ref-99] Pyburn WF (1992). A new tree frog of the genus *Scinax* from the Vaupes River of northwestern Brazil. Texas Journal of Science.

[ref-100] Pyburn WF (1993). A new species of dimorphic tree frog, genus *Hyla* (Amphibia: Anura: Hyldiae), from the Vaupés River of Colombia. Proceedings of the Biological Society of Washington.

[ref-101] Pyburn WF, Fouquette MJ (1971). A new striped treefrog from central Colombia. Journal of Herpetology.

[ref-102] R Core Team (2016). http://www.r-project.org/.

[ref-103] Randrianiaina RD, Strauß A, Glos J, Glaw F, Vences M (2011). Diversity, external morphology and ‘reverse taxonomy’ in the specialized tadpoles of Malagasy river bank frogs of the subgenus *Ochthomantis* (genus *Mantidactylus*). Contributions to Zoology.

[ref-104] Ribeiro JELS, Hopkins MJG, Vicentini A, Sothers CA, Costa MAS, Brito JM, Souza MAD, Martins LH, Lohmann LG, Assunção PA, Pereira EC, Silva CF, Mesquita MR, Procópio LC (1999). Flora da Reserva Ducke. Guia de identificação das plantas vasculares de uma floresta de terra firme na Amazônia Central.

[ref-105] Ribeiro JW, Lima AP, Magnusson WE (2012). The effect of riparian zones on species diversity of frogs in Amazonian forests. Copeia.

[ref-106] Rivero JA (1961). Salientia of Venezuela. Bulletin of the Museum of Comparative Zoology.

[ref-107] Rojas-Ahumada DP, Landeiro VL, Menin M (2012). Role of environmental and spatial processes in structuring anuran communities across a tropical rain forest. Austral Ecology.

[ref-108] Ron SR, Venegas PJ, Toral E, Read M, Ortiz DA, Manzano AL (2012). Systematics of the *Osteocephalus buckleyi* species complex (Anura: Hylidae) from Ecuador and Peru. ZooKeys.

[ref-109] Rossetti DF, Toledo PM, Góes AM (2005). New geological framework for Western Amazonia (Brazil) and implications for biogeography and evolution. Quaternary Research.

[ref-110] Santana DJ, Costa HC, Drummond LO, Ferreira PL, Feio RN (2009). Amphibia, Anura, Hylidae, *Scinax auratus*: distribution extension, new state records, and distribution map. Check List. Journal of Species Lists and Distribution.

[ref-111] Savage JM (2002). The amphibians and reptiles of Costa Rica.

[ref-112] Savage JM, Heyer WR (1967). Variation and distribution in the tree-frog genus *Phyllomedusa* in Costa Rica, Central America. Beiträge zur Neotropischen Fauna.

[ref-113] Schietti J, Emilio T, Rennó CD, Drucker DP, Costa FRC, Nogueira A, Baccaro FB, Figueiredo F, Castilho CV, Kinupp V, Guillaumet JL, Garcia ARM, Lima AP, Magnusson WE (2014). Vertical distance from drainage drives floristic composition changes in an Amazonian rainforest. Plant Ecology & Diversity.

[ref-114] Schietti J, Martins D, Emilio T, Souza PF, Levis C, Baccaro FB, Pinto JLPV, Moulatlet GM, Stark SC, Sarmento K, Araújo RNO, Costa FRC, Schöngart J, Quesada CA, Saleska SR, Tomasella J, Magnusson WE (2016). Forest structure along a 600 km transect of natural disturbances and seasonality gradients in central-southern Amazonia. Journal of Ecology.

[ref-115] Souza JP, Baccaro FB, Landeiro VL, Franklin E, Magnusson WE, Pequeno PACL, Fernandes IO (2016). Taxonomic sufficiency and indicator taxa reduce sampling costs and increase monitoring effectiveness for ants. Diversity and Distribution.

[ref-116] Stuart SN, Chanson JS, Cox NA, Young BE, Rodrigues ASL, Fischman DL, Waller RW (2004). Status and trends of amphibian declines and extinctions worldwide. Science.

[ref-117] Sturaro MJ, Peloso PLV (2014). A new species of *Scinax* Wagler, 1830 (Anura: Hylidae) from the middle Amazon River Basin, Brazil. Papéis Avulsos de Zoologia.

[ref-118] Tsuji-Nishikido BM, Menin M (2011). Distribution of frogs in riparian areas of an urban forest fragment in Central Amazonia. Biota Neotropica.

[ref-119] Vasconcelos HL, Macedo ACC, Vilhena JMS (2003). Influence of topography on the distribution of ground-dwelling ants in an Amazonian forest. Studies on Neotropical Fauna and Environment.

[ref-120] Zeileis A, Kleiber C, Jackman S (2008). Regression models for count data in R. Journal of Statistical Software.

