# Supplementary material for: A new species of Amazonian snouted treefrog (Hylidae: Scinax) with description of a novel species-habitat association for an aquatic breeding frog
Source: PeerJ. 2018 Feb 9;6:e4321. doi: 10.7717/peerj.4321 (PMC5808318; doi:10.7717/peerj.4321)
Supplement: Table S1 [file peerj-06-4321-s001.pdf]

S. baumgardneri	(Rivero, 1961)
S. blairi	(Fouquette and Pyburn, 1972)
S. boesemani	(Goin, 1966)
S. chiquitanus	(De la Riva, 1990)
S. cruentomma	(Duellman, 1972)
S. danae	(Duellman, 1986)
S. exiguus	(Duellman, 1986)
S. funereus	(Cope, 1874)
S. fuscomarginatus	(Lutz, 1925)
S. fuscovarius	(A. Lutz, 1925)
S. garbei	(Miranda-Ribeiro, 1926)
S. ictericus	Duellman and Wiens, 1993
S. iquitorum	Moravec, Tuanama, Pérez and Lehr, 2009
S. jolyi	Lescure and Marty, 2000
S. karenanneae	(Pyburn, 1992)
S. kennedyi	(Pyburn, 1973)
S. lindsayi	Pyburn, 1992
S. madeirae	(Bokermann, 1964)
S. nebulosus	(Spix, 1824)
S. onca	Ferrão, Moravec, Fraga, Almeida, Kaefer, and Lima, 2017
S. oreites	Duellman and Wiens, 1993
S. pedromedinae	(Henle, 1991)
S. proboscideus	(Brongersma, 1933)
S. rostratus	(Peters, 1863)
S. ruber	(Laurenti, 1768)
S. sateremawe	Sturaro and Peloso, 2014
S. villasboasi	Brusquetti, Jansen, Barrio-Amorós, Segalla, and Haddad, 2014
S. wandae	(Pyburn and Fouquette, 1971)
S. x-signatus	(Spix, 1824)

Supplemental table 1. Species of *Scinax* occurring in Amazonia according Sturaro & Peloso (2014) (and their references) and Ferrão et al. (2016) (and their references).
