# Supplementary material for: A new species of Amazonian snouted treefrog (Hylidae: Scinax) with description of a novel species-habitat association for an aquatic breeding frog
Source: PeerJ. 2018 Feb 9;6:e4321. doi: 10.7717/peerj.4321 (PMC5808318; doi:10.7717/peerj.4321)
Supplement: Appendix S1 — Abbreviations: (AM) Highway at State of Amazonas, Brazil; (PDBFF) Projeto Dinâmica Biológica de Fragmentos Florestais (a project in Brazil focused on dynamics of forest fragments), (km) kilometre; (INPA-H) Herpetological Section of the Zoological Collection of the Instituto Nacional de Pesquisas da Amazônia, Manaus, Brazil; (RMNH) Nationaal Natuurhistorisch Museum, Leiden, The Netherlands; (QCAZ) Museo de Zoología, Pontificia Universidad Católica del Ecuador, Quito, Ecuador; (KU) University of Kansas, Museum of Natural History, Division of Herpetology, Lawrence, Kansas, USA; (ANDES-A) Museo de Historia Natural ANDES, Universidad de los Andes, Bogotá, Colombia; (ZFMK) Zoologisches Forschungsinstitut und Museum Alexander Koenig, Herpetologische Abteilung, Adenauerallee, Germany. [file peerj-06-4321-s002.docx]

**APPENDIX 1.** **Specimens examined for morphological comparisons.**

Abbreviations: (AM) Highway at State of Amazonas, Brazil; (PDBFF) Projeto Dinâmica Biológica de Fragmentos Florestais (a project in Brazil focused on dynamics of forest fragments), (km) kilometre; (INPA-H) Herpetological Section of the Zoological Collection of the Instituto Nacional de Pesquisas da Amazônia, Manaus, Brazil; (RMNH) Nationaal Natuurhistorisch Museum, Leiden, The Netherlands; (QCAZ) Museo de Zoología, Pontificia Universidad Católica del Ecuador, Quito, Ecuador; (KU) University of Kansas, Museum of Natural History, Division of Herpetology, Lawrence, Kansas, USA; (ANDES-A) Museo de Historia Natural ANDES, Universidad de los Andes, Bogotá, Colombia; (ZFMK) Zoologisches Forschungsinstitut und Museum Alexander Koenig, Herpetologische Abteilung, Adenauerallee, Germany.

***Scinax* sp. 1**: BRAZIL: Amazonas: Tapauá (INPA-H 34688–34692, 34700).

***Scinax*** **sp. 2**: BRAZIL: Amazonas: Humaitá (INPA-H 34651, 34657, 34664, 34666–34678).

***Scinax* sp. 4**: BRAZIL: Amazonas: Humaitá (INPA-H 34693).

***Scinax* sp. 5**: BRAZIL: Amazonas: Tapauá (INPA-H 34632, 34639–34640, 34648, 34656); Berurí (INPA-H 34693, 34696, 34703); Borba (INPA-H 34710).

***Scinax*** **sp. 6**: BRAZIL: Amazonas: Careiro da Várzea, Ramal do Purupuru (INPA-H 34597); Rondônia: Porto Velho (INPA-H 35559, 35561–35568).

***Scinax boesemani***: SURINAME: Paramaribo: near Zanderij (RMNH 12601, holotype, photo). BRAZIL: Roraima: Caracaraí, Viruá National Park (INPA-H 25972, 25974).

***Scinax chiquitanus***: BRAZIL: Rondônia: Porto Velho (INPA-H 35554–35558, 35560).

***Scinax cruentomma***: ECUADOR: Napo: Santa Cecilia (KU 126587, holotype, photo); Orellana: Parque Nacional Yasuní (QCAZ 8184), Río Napo (QCAZ 43772, 44754). BRAZIL: Amazonas: Careiro da Várzea, Ramal do Purupuru (INPA-H 34697).

***Scinax* aff. *cruentomma***: BRAZIL: Amazonas: Manicoré (INPA-H 34596).

***Scinax funereus***: ECUADOR: Orellana: Río Napo, Primavera (QCAZ 43799, photo), Tambococha (QCAZ 55280, 55283; photo). PERU: Loreto: San Jacinto (KU221960b).

***Scinax fuscomarginatus***: BRAZIL: Roraima: Boa Vista, Maracá Ecological Station (INPA-H 34634, 34646, 34661–34662); Caracaraí, Viruá National Park (INPA-H 19371–19372, 19376, 19378, 19383–19384).

***Scinax garbei***: BRAZIL: Roraima: Caracaraí, Viruá National Park (INPA-H 25964, 27496).

***Scinax*** cf. ***ictericus***: PERU: Madre de Dios: Rio Tambopata (ZFMK 39353, ZFMK 39361, ZFMK 39363, ZFMK 39366).

***Scinax iquitorum***: PERU: Loreto: ca. 17 km straight SW of Iquitos, (NMP6V 71267/1‑3; paratypes).

***Scinax madeirae***: BRAZIL: Rondônia: Alta Floresta, Corumbiaria Park (INPA-H 7050–7051).

***Scinax nebulosus***: BRAZIL: Pará: Alter do Chão (INPA-H 34647, 34653); Rondônia: Costa Marques, Real Forte Príncipe da Beira (INPA-H 34641); Roraima: Caracaraí, Parque Nacional do Viruá (INPA-H 27535–27537).

***Scinax*** ***onca***: BRAZIL: Amazonas: Berurí (INPA-H 20582, 20586, 34581, 34583–34585, 34587); Rondônia: Porto Velho (INPA-H 34588–34595).

***Scinax pedromedinae***: BOLIVIA: Beni: 5 km NE of Riberalta (NMP6V 70700); PERU: Ucayali: Masisea (NMP6V 74902/1–3).

***Scinax proboscideus***: BRAZIL: Amazonas: Manaus, Colosso Reserve at PDBFF (INPA-H 10304); Presidente Figueiredo, Vila Pitinga (INPA-H 1870); Pará: Oriximiná (INPA-H 304).

***Scinax wandae***: COLOMBIA: Meta, San Juan de Arama (ANDES-A 1287, 1814–1815: photo).
