# Supplementary material for: A new species of Amazonian snouted treefrog (Hylidae: Scinax) with description of a novel species-habitat association for an aquatic breeding frog
Source: PeerJ. 2018 Feb 9;6:e4321. doi: 10.7717/peerj.4321 (PMC5808318; doi:10.7717/peerj.4321)
Supplement: Appendix S2 [file peerj-06-4321-s003.docx]

**APPENDIX 2.** **Sampling methods for the environmental variables.**

Number of trees was measured in four size classes along the 55 plots (five in each sampling module): (1) number of trees and palms with a diameter at breast height (DBH) < 10 cm, measured in the range of 1 x 250 m on the left side of the centerline of the plot; (2) number of trees and palms with DBH 10 – 20 cm, measured in the range of 20 x 250 meters, being ten meters on each side of the centerline of the plot; (3) number of trees and palms with DBH 20 – 30 cm, measured in the range of 40 x 250 m, being 20 m on each side of the centerline of the plot; (4) number of trees and palms with DBH > 30 cm, measured in the range of 40 x 250 m, being 20 m on each side of the centerline of the plot. We used the sum of four size classes to represent forest structure.

Soil samples were collected at a depth between 0 and 10 cm every 50 m along each plot and were mixed to generate a composite sample per plot. The soil samples were air-dried and the root remnants were removed. The organic matter was removed through oxidation using a treatment based on distilled water and hydrogen peroxide. The contents of silt, sand and clay were quantified. The percentage of sand was determined using a 0.053 mm mesh sieve. The proportion of clay was determined by separating the particles of 20 μm and subsequently drying them in an oven at 105 °C. The proportion of silt was determined as the complement of the percentages of clay and sand.

The depth of the water table was measured manually in five plots per sampling module through seven meters’ deep wells that were drilled at the beginning of each plot. Depth of the water table was taken during the months of March, July and November 2011, March, August, October and December 2012 and March 2013. We used the average of measurements to represent depth of water table.
