# Supplementary material for: A new species of Amazonian snouted treefrog (Hylidae: Scinax) with description of a novel species-habitat association for an aquatic breeding frog
Source: PeerJ. 2018 Feb 9;6:e4321. doi: 10.7717/peerj.4321 (PMC5808318; doi:10.7717/peerj.4321)
Supplement: Appendix S3 — Abbreviations: (INPA-H) Herpetological Section of the Zoological Collection of the Instituto Nacional de Pesquisas da Amazônia, Manaus, Brazil, (RAPELD) sampling sites, (M) male, (F) female, (SVL) snout-vent length, (HL) head length, (HW) head width, (ED) horizontal eye diameter, (UEW) upper eyelid width, (IND) internarial distance, (IOD) interorbital distance, (TD) horizontal tympanum diameter, (TL) tibia length, (FL) foot length, (END) eye-nostril distance, (3FD) third finger disk diameter, (4TD) fourth toe disk diameter, (TAL) tarsus length, (HAL) hand length, (THL) thigh length. [file peerj-06-4321-s004.docx]

**APPENDIX 3.** **Morphometric measurements of *Scinax rubrooculata* sp. nov. from Purus-Madeira Interfluve, State of Amazonas, Brazil.** Abbreviations: (INPA-H) Herpetological Section of the Zoological Collection of the Instituto Nacional de Pesquisas da Amazônia, Manaus, Brazil, (RAPELD) sampling sites, (M) male, (F) female, (SVL) snout-vent length, (HL) head length, (HW) head width, (ED) horizontal eye diameter, (UEW) upper eyelid width, (IND) internarial distance, (IOD) interorbital distance, (TD) horizontal tympanum diameter, (TL) tibia length, (FL) foot length, (END) eye-nostril distance, (3FD) third finger disk diameter, (4TD) fourth toe disk diameter, (TAL) tarsus length, (HAL) hand length, (THL) thigh length.

| INPA-H | RAPELD | Sex | SVL | HL | HW | ED | TD | UEW | IOD | IND | TAL | FL | HAL | 3FD | 4TD | END | TL | THL |
| --- | --- | --- | --- | --- | --- | --- | --- | --- | --- | --- | --- | --- | --- | --- | --- | --- | --- | --- |
| 34598 | 2 | M | 25.5 | 9.0 | 8.5 | 3.3 | 1.4 | 2.4 | 2.6 | 1.8 | 6.7 | 10.0 | 6.7 | 1.1 | 1.0 | 3.0 | 13.1 | 11.9 |
| 34599 | 7 | M | 24.8 | 8.6 | 8.3 | 2.8 | 1.4 | 2.1 | 2.2 | 1.8 | 6.5 | 9.2 | 6.1 | 0.8 | 0.8 | 2.7 | 12.4 | 11.4 |
| 34601 | 2 | M | 24.0 | 8.8 | 8.1 | 3.0 | 1.3 | 2.6 | 2.3 | 1.9 | 6.5 | 9.9 | 6.4 | 1.1 | 1.1 | 2.9 | 11.9 | 11.8 |
| 34602 | 3 | M | 22.6 | 8.3 | 7.9 | 2.8 | 1.4 | 1.8 | 2.2 | 1.7 | 6.4 | 8.6 | 6.0 | 0.9 | 0.9 | 2.4 | 11.7 | 11.0 |
| 34603 | 8 | M | 23.3 | 8.5 | 8.0 | 3.0 | 1.4 | 2.2 | 2.2 | 1.8 | 6.4 | 9.4 | 6.2 | 1.0 | 1.0 | 2.7 | 11.7 | 10.5 |
| 34604 | 2 | M | 25.8 | 9.5 | 8.5 | 3.0 | 1.6 | 2.4 | 2.5 | 1.8 | 6.7 | 9.9 | 6.7 | 0.8 | 0.9 | 3.0 | 12.7 | 11.4 |
| 34608 | 5 | M | 23.6 | 8.6 | 7.9 | 2.8 | 1.5 | 2.5 | 2.4 | 1.9 | 6.5 | 9.5 | 6.2 | 0.8 | 0.8 | 2.8 | 11.7 | 11.4 |
| 34609 | 7 | M | 24.5 | 9.1 | 8.7 | 3.4 | 1.6 | 2.5 | 2.4 | 1.8 | 6.6 | 9.0 | 6.3 | 0.8 | 0.9 | 2.8 | 12.1 | 11.3 |
| 34610 | 4 | M | 23.1 | 8.8 | 8.2 | 2.7 | 1.2 | 2.4 | 2.3 | 1.8 | 6.0 | 8.9 | 5.9 | 0.9 | 0.9 | 2.6 | 11.6 | 11.0 |
| 34611 | 7 | M | 23.8 | 9.7 | 8.3 | 3.0 | 1.3 | 2.4 | 2.5 | 1.8 | 6.7 | 9.9 | 6.5 | 1.1 | 1.0 | 3.0 | 12.4 | 11.3 |
| 34612 | 7 | M | 24.4 | 9.3 | 8.6 | 3.1 | 1.4 | 2.6 | 2.4 | 1.9 | 6.8 | 10.3 | 7.0 | 0.8 | 0.8 | 3.0 | 12.7 | 12.0 |
| 34613 | 9 | M | 24.7 | 9.3 | 8.8 | 3.0 | 1.4 | 2.2 | 2.4 | 1.8 | 7.0 | 9.8 | 6.7 | 1.2 | 1.2 | 3.0 | 12.7 | 11.5 |
| 34614 | 2 | M | 25.5 | 8.6 | 8.7 | 3.0 | 1.5 | 2.5 | 2.5 | 1.7 | 6.6 | 10.3 | 6.4 | 1.1 | 1.1 | 2.6 | 12.6 | 11.8 |
| 34615 | 2 | M | 24.6 | 8.8 | 8.5 | 3.2 | 1.5 | 2.5 | 2.3 | 1.7 | 6.5 | 9.6 | 6.6 | 1.1 | 1.1 | 2.8 | 12.1 | 11.5 |
| 34616 | 8 | M | 24.2 | 8.3 | 8.1 | 3.0 | 1.3 | 2.4 | 2.3 | 1.8 | 6.7 | 9.3 | 6.1 | 0.8 | 0.8 | 2.7 | 12.2 | 11.4 |
| 34617 | 7 | M | 23.7 | 9.0 | 8.2 | 2.8 | 1.2 | 2.1 | 2.3 | 1.9 | 6.4 | 9.2 | 6.4 | 0.9 | 1.0 | 2.8 | 12.1 | 11.5 |
| 34618 | 7 | M | 25.2 | 8.7 | 8.6 | 2.8 | 1.2 | 2.1 | 2.5 | 1.7 | 6.8 | 9.8 | 6.6 | 1.0 | 1.1 | 2.9 | 12.5 | 11.4 |
| 34619 | 9 | M | 24.9 | 9.0 | 8.6 | 3.2 | 1.6 | 2.3 | 2.2 | 1.8 | 6.6 | 9.8 | 6.6 | 1.1 | 1.3 | 2.9 | 12.3 | 10.2 |
| 34621 | 7 | M | 25.7 | 9.8 | 8.9 | 3.2 | 1.5 | 2.5 | 2.4 | 1.9 | 7.0 | 10.1 | 6.8 | 0.9 | 0.9 | 3.1 | 13.1 | 12.1 |
| 34622 | 2 | M | 24.5 | 8.8 | 8.4 | 3.4 | 1.5 | 2.3 | 2.2 | 1.9 | 6.8 | 9.6 | 6.8 | 1.1 | 0.8 | 2.9 | 12.3 | 12.1 |
| 34623 | 8 | M | 23.9 | 8.7 | 8.3 | 2.9 | 1.4 | 2.3 | 2.4 | 1.6 | 6.6 | 9.0 | 6.3 | 0.8 | 0.9 | 2.7 | 12.0 | 11.5 |
| 34624 | 2 | M | 24.2 | 8.7 | 8.3 | 3.1 | 1.4 | 2.4 | 2.5 | 2.0 | 6.5 | 9.8 | 6.2 | 0.7 | 1.0 | 2.9 | 12.5 | 11.9 |
| 34625 | 7 | M | 23.8 | 8.8 | 8.1 | 2.8 | 1.1 | 2.1 | 2.4 | 1.5 | 6.5 | 9.0 | 6.2 | 0.7 | 0.7 | 2.7 | 12.3 | 11.8 |
| 34626 | 7 | M | 22.9 | 8.9 | 8.4 | 3.0 | 1.4 | 1.9 | 2.4 | 1.8 | 6.2 | 9.0 | 6.3 | 0.8 | 0.8 | 2.5 | 11.4 | 10.8 |
| 34627 | 2 | M | 24.6 | 8.9 | 8.6 | 3.0 | 1.4 | 2.6 | 2.5 | 1.8 | 6.5 | 9.7 | 6.6 | 1.0 | 1.0 | 2.9 | 12.4 | 11.7 |
| 34628 | 7 | M | 25.2 | 9.7 | 8.1 | 3.2 | 1.4 | 2.5 | 2.5 | 1.8 | 6.7 | 9.9 | 6.7 | 0.9 | 0.9 | 3.0 | 12.4 | 12.1 |
| 34665 | 9 | M | 25.9 | 9.6 | 8.8 | 3.1 | 1.5 | 2.4 | 2.7 | 2.0 | 7.2 | 9.7 | 7.6 | 0.9 | 0.9 | 2.9 | 13.0 | 12.1 |
| 34629 | 2 | M | 24.1 | 8.8 | 8.1 | 2.8 | 1.3 | 2.5 | 2.2 | 1.9 | 6.5 | 9.4 | 6.4 | 1.0 | 1.1 | 2.7 | 11.2 | 11.5 |
| 34605 | 9 | F | 26.2 | 9.7 | 9.6 | 3.2 | 1.4 | 2.7 | 2.4 | 1.8 | 7.4 | 10.8 | 7.8 | 1.2 | 1.0 | 3.3 | 13.7 | 12.7 |
| 34600 | 2 | F | 27.5 | 9.7 | 9.5 | 3.2 | 1.5 | 2.6 | 2.5 | 2.0 | 7.3 | 10.5 | 7.4 | 0.9 | 1.1 | 2.9 | 13.8 | 12.5 |
| 34606 | 8 | F | 27.0 | 9.8 | 9.3 | 2.9 | 1.5 | 2.2 | 2.8 | 1.8 | 7.9 | 10.7 | 7.6 | 1.1 | 0.9 | 3.3 | 14.2 | 13.4 |
| 34630 | 7 | F | 25.8 | 9.4 | 8.9 | 2.9 | 1.6 | 2.5 | 2.6 | 2.1 | 7.2 | 10.0 | 6.8 | 0.9 | 0.8 | 3.1 | 13.3 | 12.7 |
| 34607 | 7 | F | 25.4 | 9.4 | 8.8 | 3.0 | 1.4 | 2.5 | 2.4 | 1.9 | 7.1 | 10.1 | 7.0 | 1.2 | 1.1 | 3.0 | 13.1 | 10.2 |
| 34620 | 4 | F | 25.8 | 9.4 | 8.6 | 2.9 | 1.4 | 2.3 | 2.5 | 1.9 | 7.0 | 9.5 | 6.2 | 0.9 | 0.9 | 3.0 | 12.9 | 12.0 |
