# Supplementary material for: A new species of Amazonian snouted treefrog (Hylidae: Scinax) with description of a novel species-habitat association for an aquatic breeding frog
Source: PeerJ. 2018 Feb 9;6:e4321. doi: 10.7717/peerj.4321 (PMC5808318; doi:10.7717/peerj.4321)
Supplement: Appendix S4 — Abbreviations: (PC) Principal Component, (SVL) snout-vent length, (HL) head length, (HW) head width, (ED) horizontal eye diameter, (UEW) upper eyelid width, (IND) internarial distance, (IOD) interorbital distance, (TD) horizontal tympanum diameter, (TL) tibia length, (FL) foot length, (END) eye-nostril distance, (3FD) third finger disk diameter, (4TD) fourth toe disk diameter, (TAL) tarsus length, (HAL) hand length, (THL) length thigh. [file peerj-06-4321-s005.docx]

**APPENDIX 4. Loadings resulting from the Principal Component Analyses conducted with the SVL and 15 morphometric ratios (scaled) from all individuals of *Scinax ruberoculatus* sp. nov. (males and females).** Abbreviations: (PC) Principal Component, (SVL) snout-vent length, (HL) head length, (HW) head width, (ED) horizontal eye diameter, (UEW) upper eyelid width, (IND) internarial distance, (IOD) interorbital distance, (TD) horizontal tympanum diameter, (TL) tibia length, (FL) foot length, (END) eye-nostril distance, (4FD) fourth finger disk diameter, (4TD) fourth toe disk diameter, (TAL) tarsus length, (HAL) hand length, (THL) length thigh.

|  | **PC1** | **PC2** | **PC3** | **PC4** | **PC5** | **PC6** | **PC7** | **PC8** | **PC9** | **PC10** | **PC11** | **PC12** | **PC13** | **PC14** | **PC15** | **PC16** |
| --- | --- | --- | --- | --- | --- | --- | --- | --- | --- | --- | --- | --- | --- | --- | --- | --- |
| **SVL** | -0.0893 | -0.1496 | 0.3909 | -0.2586 | 0.5376 | -0.1056 | 0.1056 | -0.1006 | 0.0975 | -0.1870 | 0.0545 | -0.4160 | 0.0686 | 0.0007 | 0.3974 | 0.2132 |
| **HL/SVL** | 0.3441 | 0.0051 | -0.2137 | 0.0545 | -0.0986 | -0.3473 | -0.0987 | -0.5343 | -0.2891 | 0.0586 | 0.1956 | -0.0191 | 0.2651 | -0.0687 | 0.3892 | -0.2352 |
| **HW/SVL** | 0.2861 | 0.0832 | 0.0570 | 0.4527 | 0.0261 | -0.4649 | 0.2130 | 0.1719 | 0.4198 | 0.0203 | 0.3106 | -0.0201 | 0.1485 | 0.0116 | -0.1763 | 0.2907 |
| **ED/SVL** | 0.1392 | 0.3383 | -0.2736 | 0.1724 | 0.3136 | -0.0491 | -0.0836 | 0.2758 | -0.3681 | 0.4141 | -0.2420 | -0.1967 | -0.2048 | -0.0776 | 0.1782 | 0.3064 |
| **TD/SVL** | -0.0126 | 0.1895 | -0.2892 | 0.2256 | 0.5878 | 0.3617 | -0.2288 | -0.0016 | 0.1479 | -0.1829 | 0.2116 | 0.0527 | 0.3037 | -0.0292 | -0.1282 | -0.2972 |
| **UEW/SVL** | 0.1364 | 0.3118 | -0.1863 | -0.5267 | 0.1413 | -0.2197 | 0.0872 | 0.1673 | 0.1163 | -0.0894 | 0.3570 | 0.2926 | -0.4199 | 0.1601 | 0.0972 | -0.1492 |
| **IOD/SVL** | 0.1912 | -0.2863 | -0.3110 | 0.0864 | -0.0362 | -0.1298 | -0.4978 | 0.0232 | 0.1063 | -0.5316 | -0.2260 | -0.0629 | -0.2513 | 0.1953 | 0.0529 | 0.2403 |
| **IND/SVL** | 0.1057 | 0.2180 | -0.3703 | 0.0096 | -0.1459 | 0.3239 | 0.4895 | -0.3507 | 0.3481 | -0.0658 | -0.1840 | -0.3274 | -0.1339 | 0.1324 | 0.0938 | 0.0671 |
| **TAL/SVL** | 0.3480 | -0.2266 | 0.1433 | 0.1971 | -0.0071 | 0.4536 | 0.1154 | -0.0124 | -0.1105 | -0.1008 | 0.3259 | 0.2291 | -0.3366 | -0.4191 | 0.2101 | 0.1865 |
| **FL/SVL** | 0.3657 | 0.2125 | 0.0626 | -0.3316 | -0.0661 | 0.0755 | -0.0449 | 0.1637 | 0.2236 | -0.0913 | -0.4140 | 0.2978 | 0.4604 | -0.2974 | 0.1528 | 0.1616 |
| **HAL/SVL** | 0.3608 | -0.0244 | 0.1980 | 0.1127 | 0.2602 | -0.1543 | 0.3836 | -0.0032 | -0.3192 | -0.3075 | -0.3786 | 0.0369 | -0.1337 | 0.0485 | -0.3128 | -0.3456 |
| **4FD/SVL** | 0.1848 | 0.3404 | 0.3673 | 0.1521 | -0.1744 | 0.2766 | -0.0979 | 0.0852 | -0.1931 | -0.1210 | 0.1177 | 0.0305 | 0.1316 | 0.6605 | 0.2019 | 0.0839 |
| **4TD/SVL** | 0.0380 | 0.4475 | 0.2735 | 0.0647 | -0.2481 | -0.0477 | -0.3048 | 0.0633 | 0.1203 | -0.1816 | 0.0357 | -0.4553 | -0.2190 | -0.4008 | -0.0239 | -0.3055 |
| **END/SVL** | 0.3900 | -0.0346 | 0.0818 | -0.3644 | 0.0657 | 0.1393 | -0.2311 | -0.2879 | -0.0618 | 0.2311 | 0.1635 | -0.2325 | 0.0169 | 0.0588 | -0.5855 | 0.2502 |
| **TL/SVL** | 0.3136 | -0.3098 | 0.1453 | 0.0629 | 0.0852 | 0.0734 | -0.1694 | 0.1022 | 0.4153 | 0.4891 | -0.1780 | -0.0321 | -0.1670 | 0.1999 | 0.2000 | -0.4201 |
| **THL/SVL** | 0.1864 | -0.2884 | -0.2649 | -0.1846 | -0.1962 | 0.0998 | 0.1799 | 0.5619 | -0.1874 | -0.0893 | 0.2283 | -0.4268 | 0.2735 | 0.0128 | 0.0288 | -0.1679 |
| **Variance (%)** | **23.01** | **16.35** | **13.78** | **8.298** | **7.55** | **5.231** | **4.953** | **4.927** | **3.797** | **3.516** | **2.148** | **2.002** | **1.675** | **1.171** | **0.917** | **0.685** |
| **Cumulative (%)** | **23.01** | **39.36** | **53.13** | **61.429** | **68.98** | **74.209** | **79.163** | **84.09** | **87.886** | **91.403** | **93.551** | **95.553** | **97.228** | **98.398** | **99.315** | **100** |
